# A novel semi-quadratic buck-boost structures with continuous input current for PV application

**DOI:** 10.1038/s41598-024-65012-5

**Published:** 2024-06-19

**Authors:** Mustafa Okati, Mahdiyeh Eslami, Baseem Khan

**Affiliations:** 1 Department of Electrical Engineering, Zabol Branch, Islamic Azad University, Zabol, Iran; 2 Department of Electrical Engineering, Kerman Branch, Islamic Azad University, Kerman, Iran; 3 Department of Electrical and Computer Engineering, Hawassa University, Hawassa, Ethiopia; 4 Center for Renewable Energy and Microgrids, Huanjiang Laboratory, Zhejiang University, Zhuji, Zhejiang 311816 China

**Keywords:** Buck/boost converter, Semi-quadratic, Continuous input/output current, Steady-state analysis, Solar PV system, Engineering, Electrical and electronic engineering

## Abstract

This paper recommends new design for non-isolated semi-quadratic buck/boost converter with two similar structure that includes the following features: (a) the continuous input current has made it reasonable for PV solar applications and reduced the value of the capacitors in the input filter reducing the input ripple as well as EMI problems; (b) the topology is simple, and consists of a few numbers of components; (c) the semiconductor-based components have lower current/voltage stresses in comparison with the recently recommended designs; (d) semi-quadratic voltage gain is D (2 − D) / (1 − D)^2^; (e) 94.6 percent from the theoretical relations and 91.8 percent from the experimental for the output power of 72W, the duty of 54.2 percent, and output voltage of 72 V are the efficiency values in boost mode; (f) 89.3 percent from the theoretical relations and 87.2 percent from the experimental for the output power of 15W, the duty of 25.8 percent, and output voltage of 15 V are the efficiency values in buck mode. One structure is the continuous output current and negative output polarity, and other structure is positive output polarity. The recommended topologies have been studied in both ideal and non-ideal modes. The continuous current mode (CCM) is the suggested mode for the proposed converters. Moreover, the requirements of CCM have been discussed. The various kinds of comparisons have been held for voltage gain, efficiency, and structural details, and the advantages of the suggested design have been presented. A small-signal analysis has been completed, and the suitable compensator has been planned. Finally, PLECS simulation results have been associated with the design considerations.

## Introduction

Nowadays, there are many issues concerning emissions and global warming that affects the environment. The main factor contributing to these problems is the procedure of fossil fuels such as oil and gas. In response, many countries are gradually using renewable energy (RE) resources instead of fossil fuels, such as hydropower, photovoltaic (PV) systems, wind energy, and fuel cells (FCs). Due to their varying magnitudes of voltage and current and their intermittent nature, these systems can only be used for a limited range of applications. As an example, when these systems are to be integrated with the grid, their voltage should be improved in order to control the grid's energy flow. On the contrary, it is necessary to reduce their voltage to utilize them as energy sources for some consumers to avoid damage caused by overvoltage^1–4^.

PV panels are the optimal choice for converting solar energy into electrical power. In order to accommodate the lower output voltage of solar panels, it is necessary to install a step-up DC converter to adjust the voltage for consumers connected to the power grid. Thus, numerous high-voltage gain DC converters have been presented for meeting such requirements^5,6^. Non-isolated fundamental DC converters were reviewed by some articles^7,8^ addressing traditional DC-DC converter usage for RE applications. However, conventional DC/DC converters have some limitations. Therefore, some insights have been provided into designing new high-gain DC converters for DC microgrids. Nevertheless, some applications involving renewable energy sources require a constant output voltage regardless of input voltage variations, such as LED lighting, portable devices, and so on. Buck-boost converters are a good choice for these situations^9–11^.

**Figure 1:**
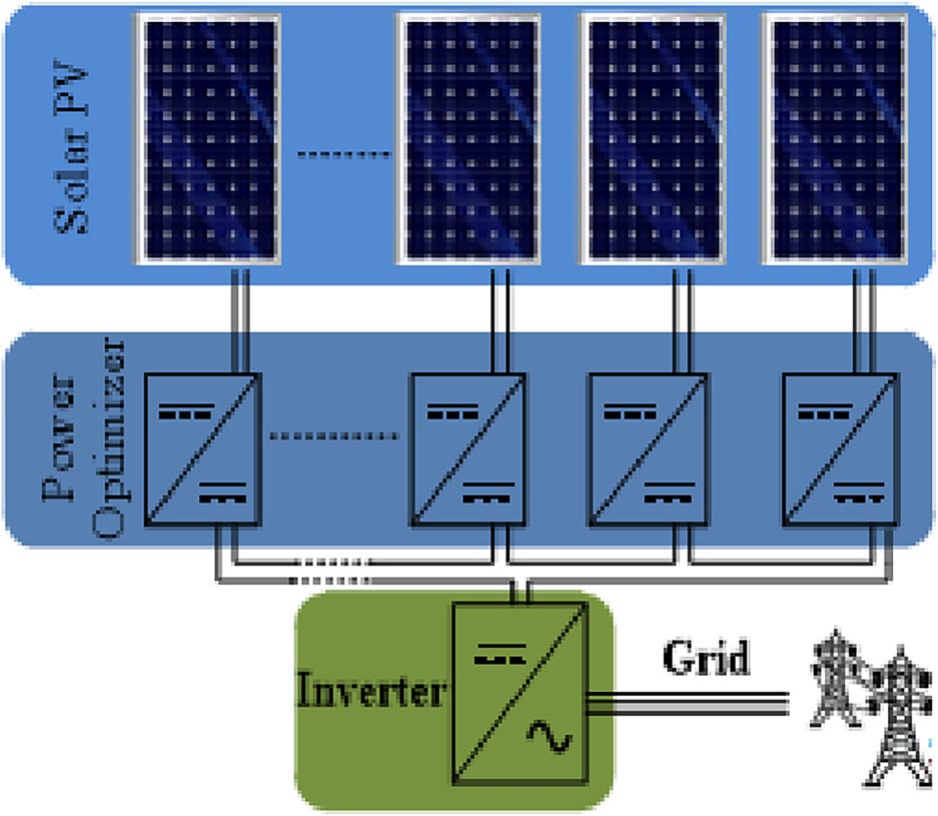
Simple Schematic of application of solar PV system with SPOs.

Many buck/boost DC converters were studied in the literature devoted to traditional DC converters, such as buck-boost, ZETA, SEPIC, CUK, buck and, boost converters^12,13^. Solar Power Optimizers (SPOs) and other applications requiring higher voltage gain can benefit from these converters^14–16^. Figure 1 represents the applications of PV solar systems with SPOs. It is evident that solar energy power can be delivered to the grid using a step-up/down converter with higher voltage gain. In situations where there is no grid connection (for instance, because the grid is redundant), it may be utilized as a buck/boost converter in uninterruptible power supply (UPS) systems for charging the lower voltage battery. As a non-isolated converter topology has no isolation among the output and input sides, the converters' output side can be directly affected by the changes on the input side. They have a lower component count than the isolated converter topology^17^. Though there are some small issues with them, which should be stated, they include poor voltage gain, higher duty cycle ratios, as well as additional circuitry for optimum performance. An analysis of some topologies mathematically has been done in order to gain a full understanding of the dynamic behavior of the converter^18^. For RE applications, SEPIC converters were utilized with various elements^19^ for lessening the voltage stress on the main switch and increasing the voltage gain. A novel buck/boost DC/DC converter was moreover given with two MOSFETs requiring two drivers. This increases the complexity of the control system along with the discontinuous inputs and outputs^20^. With a discontinuous input current port and an extreme duty cycle, this converter is twice as big as the fundamental buck/boost converter. Hence, it is not usable due to the limitations of power semiconductor tools^21^.

In^22^, proposed semi-quadratic DC converters with many elements and continuous input current port. A novel coupled-inductor buck-boost converter was presented^23^, which consists of a simple configuration with two cascade semi-stages. There are several properties of this configuration, including common ground, continuous input current and ultra-extended output voltage. The output current is discontinuous despite its enhanced performance in both boosting and bucking modes. The converter requires two floating switches. Some introduced quadratic buck/boost converters^24,25^, with continuous input current port and lower voltage gain. A novel (N/O) buck/boost converter was proposed by combining buck-boost and conventional boost converters^26^. However, there is an excessive amount of stress across one switch due to the negative and discontinuous output voltages. A boost mode and a buck/boost mode with a high gain ratio are two operating modes. Towards this end, several boosting approaches, such as the employment of voltage multipliers, switched inductors, magnetic coupling switched capacitors, and multistage techniques, are covered in^27^. Using dual working modes, a DC/DC converter with positive output displayed reduced total switching device power^28^. There were two modes of operation: step-up and step-up/down.

A N/O converter was presented with a wide conversion ratio in^29^, which suffers from sporadic input/output current. There is a critical role for N/O polarity in several industries, including the transmission of data, solar, and wind power generation. However, the discontinuous input current of the converter may have limited usage in most applications, like solar energy conversion. A prolonged multi-cell buck-boost converter was proposed based on the conventional SEPIC converter^30^, which requires several components but possesses limited voltage gain. A higher step-up buck-boost converter was also suggested^31^, which has a main switch and reduced stress around the main switch. Though, the three diodes of the converter possess high voltage stresses. A non-quadratic single switch having continuous input/output current ports was presented with no common ground connecting the input and output^32^. ZETA DC converters also exist^33^. The ZETA converter has a voltage gain twice that of the conventional ZETA converter. The design of a transformerless DC/DC converter with dual operation modes buck/boost is presented. This system acts as a common ground, continuous input current between the input and output terminals since it have a common and simple configuration^34^. Moreover, dual-mode transformerless inverters were also prototyped and presented^35^. Using this simple structure, the single-phase inverter overcomes the shortcomings of modern dual-mode inverters by providing various voltage gain ratios. There is a low voltage gain ratio in this converter. The continuous current in the output and input positive terminal polarity and the output polarities of a quadratic DC/DC converter were compared and evaluated^36^. In addition, an innovative transformerless converter with a quadratic buck-boost design has been proposed with positive output^37^. Due to the presence of inductive filters in the input and output ports, the structure has continuous input and output currents. In order to solve the discontinuity problem, a quadratic buck-boost converter was developed with a low number of elements and continuous inputs and outputs^38^. A similar study introduced an output and input inductive filter buck-boost converter^39^. The switch used in this converter has a lower voltage stress. However, this converter includes a greater number of semiconductor elements than the other structures. The ripple of the output voltage of the quadratic gain converter^40^ at the selected operating point is zero. However, high voltage stress across the output diode, this converter transmits negative voltage to output. The converter's quadratic output voltage gain^41^, in comparison to traditional buck-boost converters, allows it to achieve improved buck or boost capabilities when the duty cycle is either higher or lower than 50 percent. This converter has advanced features including input and output current ports that provide a smooth flow of electricity, reducing the fluctuations in both the input and output. As a result, it is a suitable option for applications in RE. Three different quadratic buck DC-DC converter schemes were presented, each with the ability to flip to a semi-quadratic buck-boost configuration. The topology introduced earlier in^42^ provides a cascaded structure where two similar boost converters are connected back-to-back to increase the output voltage. However, the numerous components in this design increase the size and cost. The proposed converter^43^ is described in detail. It presents a novel high-voltage gain converter that utilizes the asymmetric input voltage of inductors. This converter offers high power density, desirable output, and constant input, making it suitable for renewable energy applications. Additionally, cascaded boost converters^44^ with quadratic gain characteristics are proposed, where component count is minimized and voltage stress on passive devices is reduced. However, the voltage gain remains lower in one such model compared to another. Furthermore, both models require separate control grounds for semiconductor switches, necessitating two control power supplies. A non-isolated quadratic boost DC converter is introduced^45^, which provides high output voltage gain with a lower number of components. An input filter design makes the converter suitable for photovoltaic applications. Notably, this converter utilizes a single switch ground, eliminating the need for an additional control power supply. In a converter described in^46^ , the power switch experiences a high voltage equal to the output voltage, which distributes the voltage stress across two power switches and lowers the voltage stress on the switches. Numerous studies have proposed improved architectures for quadratic boost converters due to their flexibility for new combinations, continuous input current, the presence of ground between the source and load, and clear voltage gain for the foundation of new configurations. A topological structure is established to ensure a secure transition from a particular switch to a different one, while preserving the functional contribution of all other components towards achieving efficient power conversion, as documented in^47^. A non-isolated DC buck boost converter with low element count and low electrical stress on the elements is presented^48^. But the voltage gain ratio of this converter is very low.

The Cuk converter is the only common converter achieving continuous outputs and inputs current with a lower number of components (one switch, one capacitor, one diode, and two inductors) and flexible output. However, it has output voltage inversion. Due to its step-up/step-down capability, the Cuk converter is extensively utilized in power electronic applications. As long as the continuous output current is taken into account, parallel connections of the converter output can be made to a voltage source with negligible shunt capacitance. Utilizing larger inductors and a higher switching frequency can minimize the input current ripple problem. However, bulky filters are required by these conventional solutions; thus, they are expensive and require larger sizes along with higher switching losses. Consequently, the proposed method utilizes minimal filtering to provide a low-input current ripple converter. In this paper, two converters are proposed for PV sources to reduce input current ripple, thereby maximizing overall efficiency (while avoiding coupling an inductor). Moreover, one of semi-quadratic buck/boost converters are presented that overcomes the deficiencies of a conventional Cuk converter. It has extensive conversion ratios, as well as a continuous input/output current port. Therefore, it is appreciate for requests involving RE.

The suggested paper is organized as below. The semi-quadratic buck-boost structures and its operation, efficiency analysis and power loss analysis are described in Sect. “Proposed structuers, operation principles, and steady-state evaluation”. Section “Analysis and design parameters” analysis and design of parameters inductor and capacitor. In Sect. “Small signal modeling”, small signal modeling is evaluated. Section “Comparison with existing converters” deals with the benefits of the proposed converter in comparison with existing converters. The PLECS simulation and experimental results are shown in Sect. “Experimental result and building a prototype”. Furthermore, Sect. “Building a prototype and performance evaluation” shows the investigational outcomes of the proposed structures prototype. Finally, Sect. “Conclusion” concludes the paper with the main findings.

## Proposed structuers, operation principles, and steady-state evaluation

### Proposed semi-quadratic buck-boost converters

The most important characteristics of a DC buck/boost converter utilized by solar PV systems are constant input/output current port, cost-effectiveness, high efficiency and low noise.

**Figure 2:**
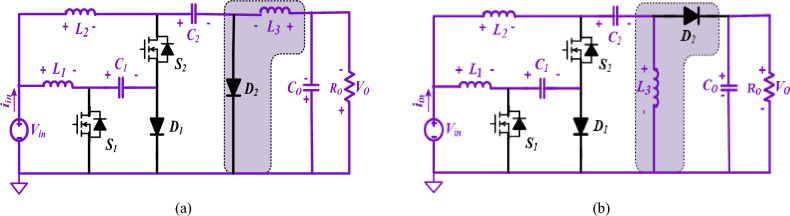
Semi-quadratic proposed converters. (**a**) Structure1, (**b**) Structure2.

Figure 2 shows the developed two similar semi-quadratic buck/boost converters. Three capacitors (*C*_*1*_*, C*_*2*_*, C*_*o*_) and three inductors (*L*_*1*_*, L*_*2*_*, L*_*3*_) and, two switches (*S*_*1*_*, S*_*2*_), two diodes (*D*_*1*_*, D*_*2*_), and a resistive load (*R*_*o*_) are combined to attain the high-voltage gain buck/boost converter.

**Figure 3:**
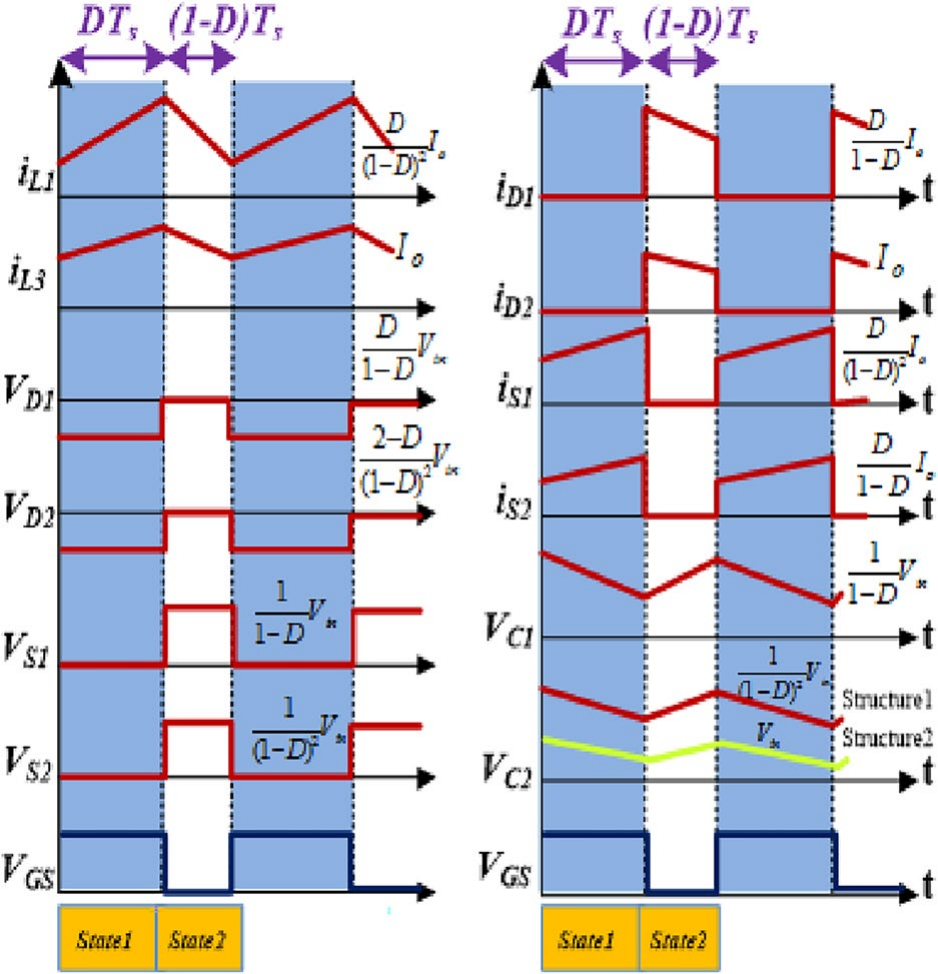
Waveforms related with the converters in CCM (Structure1and 2).

By considering the ideal components and then taking all capacitors with the appropriate size to keep nearly constant voltages, the steady-state analysis for the converter can be simplified. As a result, the voltage and current were assumed to be constant for the duration of a full period. In addition, Fig. 3 includes some graphs pertaining to converters, such as diode voltages, switch voltages, inductor currents, etc.

### States of operation principles

**Figure 4:**
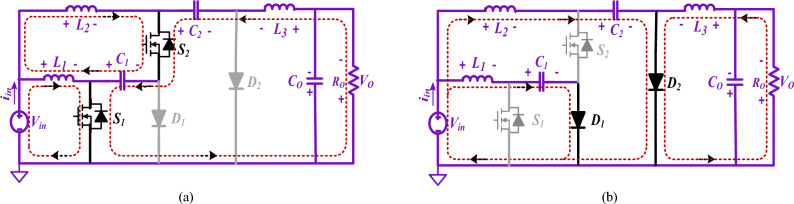
States of operation of the suggested converter for buck/boost mode (Structure1). (**a**) State 1(switches are ON); (**b**) State 2(switches are OFF).

**Figure 5:**
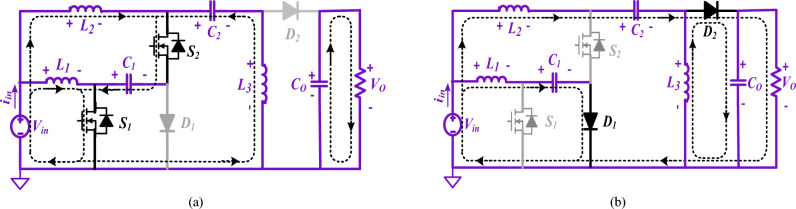
States of operation of the suggested converter for buck/boost mode (Structure2). (**a**) State 1(switches are ON); (**b**) State 2(switches are OFF).

It is necessary to take into consideration both steady-state conditions as well as CCM when calculating the characteristics of the proposed converters. Thus, the consistent current and voltage are considered over a complete switching duration. According to Figs. 4 and 5, the converter in the CCM can operate in two major modes.

#### Structure1

The inductors *L*_*3*_ and *L*_*1*_ in the output and input terminals of the suggested converter ensure output/input current continuity.

##### State 1: When switches are on [0 ≤ t ≤ DTs]

The circuit operates in this state if power switches *S*_2_ and *S*_*1*_ are ON while two diodes *D*_2_ and *D*_*1*_ are in reversed-biased mode (Fig. 4(a)). The inductors *L*_*1*_*, L*_*2*_, and*, L*_*3*_ are powered by the input source *V*_*in*_ through the capacitor *C*_*1*_. At this stage, the capacitors *C*_*1*_ and *C*_*2*_ are depleted, resulting in an escalation of current in the inductors (*i*_*L1*_*, i*_*L2*_*, i*_*L3*_). The load impedance *R*_*o*_ is supplied by the output capacitor *C*_*o*_ (Fig. 4a). Using the KCL and KVL, the current and voltage equations are achieved.

1 $$\left\{ {\begin{array}{*{20}c} {V_{L1} = V_{in} ,V_{L2} = V_{in} + V_{C1} ,V_{L3} = V_{C1} + V_{C2} - V_{o} } \\ {i_{C1} = - i_{L3} - i_{L2} ,i_{C2} = - i_{L3} ,i_{Co} = - i_{L3} - \left( {V_{o} /R_{o} } \right)} \\ \end{array} } \right.$$

##### State 2: When switches are off [DTs ≤ t ≤ Ts]

In this state, no power is being supplied by either power switch. Moreover, the capacitors *C*_1_ and *C*_2_ are charged, the inductors *L*_1_, *L*_2_, and *L*_3_ are demagnetized, and the two diodes *D*_2_ and *D*_1_ are in conduction mode (Fig. 4b). It should be noted that the inductor *L*_3_ feeds the output load *R*_*o*_ and also charges *C*_*o*_. By the current path of *D*_1_ and *D*_2_ diodes, energy is discharged from capacitors *C*_1_ and *C*_*2*_. In this state, through the KVL and KCL, the voltage and current calculations are realized.

2 $$\left\{ {\begin{array}{*{20}c} {V_{L1} = V_{in} - V_{C1} ,V_{L2} = V_{in} - V_{C2} ,V_{L3} = - V_{o} } \\ {i_{C1} = i_{L1} ,i_{C2} = i_{L2} + i_{L3} ,i_{Co} = - i_{L3} - \left( {V_{o} /R_{o} } \right)} \\ \end{array} } \right.$$

#### Structure2

In this structure, the position of output inductor and diode has been changed; as a result, discontinuous current output port with positive output polarity has been created.

##### State 1: When switches are on [0 ≤ t ≤ DTs]

The circuit is in this state when power switches *S*_2_ and *S*_*1*_ are turned ON, while two diodes *D*_2_ and *D*_*1*_ are in a reversed-biased mode (Fig. 5a). The inductors *L*_*1*_*, L*_*2*_, and*, L*_*3*_ are powered by the input source *V*_*in*_ through the capacitor *C*_*1*_. At this stage, the capacitors *C*_*1*_ and *C*_*2*_ are completely drained, leading to an increase in the current flowing through the inductors (*i*_*L1*_*, i*_*L2*_*, i*_*L3*_). The load resistance Ro is supplied by the output capacitor *C*_*o*_ (Fig. 5a). Using the KCL and KVL, the current and voltage equations are achieved.

3 $$\left\{ {\begin{array}{*{20}c} {V_{L1} = V_{in} ,V_{L2} = V_{in} + V_{C1} ,V_{L3} = - V_{C1} - V_{C2} } \\ {i_{C1} = - i_{L2} - i_{L3} ,i_{C2} = i_{L3} ,i_{Co} = - \left( {V_{o} /R_{o} } \right)} \\ \end{array} } \right.$$

##### State 2: When switches are off [DTs ≤ t ≤ Ts]

In this state, no power is being supplied by either power switch. Moreover, the capacitors *C*_1_ and *C*_2_ are stimulating, the inductors *L*_1_, *L*_2_, and *L*_3_ are demagnetized, and the two diodes *D*_2_ and *D*_1_ are in conduction mode (Fig. 5b). It should be noted that the inductor *L*_3_ feeds the output load *R*_*o*_ and also charges *C*_*o*_. By the current path of *D*_1_ and *D*_2_ diodes, energy is discharged from capacitors *C*_1_ and *C*_*2*_. In this state, through the KCL and KVL, the current and voltage equations are achieved,

4 $$\left\{ {\begin{array}{*{20}c} {V_{L1} = V_{in} - V_{C1} ,V_{L2} = V_{in} - V_{C2} - V_{o} ,V_{L3} = V_{o} } \\ {i_{C1} = i_{L1} ,i_{C2} = i_{L2} ,i_{Co} = + i_{L2} - i_{L3} - \left( {V_{o} /R_{o} } \right)} \\ \end{array} } \right.$$

### Steady-state evaluation (structure1 and 2)

The gain ratios of voltage and current relations of the introduced converters presented in Figs. 4 and 5 (State 1 & 2) are derived in this section. With the volt-second balance principle of inductors *L*_*1*_, *L*_*2*_, and *L*_*3*_, the average capacitor voltages *C*_*1*_ and *C*_*2*_:

5 $$\begin{array}{*{20}c} { Structure1\left\{ {V_{C1} = \frac{1}{{\left( {1 - D} \right)}}V_{in} ,V_{C2} = \frac{1}{{\left( {1 - D} \right)^{2} }}V_{in} } \right.} \\ {Structure2\left\{ {V_{C1} = \frac{1}{{\left( {1 - D} \right)}}V_{in} ,V_{C2} = V_{in} } \right.} \\ \end{array}$$

Therefore, the voltage gain is obtained as,

6 $$\begin{array}{*{20}c} {Structure1\left\{ {M_{CCM} = \left( {\frac{{V_{o} }}{{V_{in} }}} \right) = - \frac{{D\left( {2 - D} \right)}}{{\left( {1 - D} \right)^{2} }}} \right.} \\ {Structure2\left\{ {M_{CCM} = \left( {\frac{{V_{o} }}{{V_{in} }}} \right) = + \frac{{D\left( {2 - D} \right)}}{{\left( {1 - D} \right)^{2} }}} \right.} \\ \end{array}$$

The relations obtained below are the same for both structures. Considering there is no circuit loss, the powers of input and output can be calculated by:

7 $$P_{in} = P_{o} ,{ }P_{in} = { }V_{in} I_{in} ,P_{o} = V_{o} I_{o}$$

The current gain is attained based on (6):

8 $$\frac{{I_{o} }}{{I_{in} }} = \frac{{\left( {1 - D} \right)^{2} }}{{D\left( {2 - D} \right)}}$$

Using the ampere-second balance principle, the mean current value of all inductor currents is defined by the capacitance* C*_*o*_, *C*_*2*_, and* C*_*1*_.

9 $$I_{L3} = \left| {I_{o} } \right|, I_{L2} = \frac{{D\left| {I_{o} } \right|}}{1 - D},{ } I_{LI} = \frac{D}{{\left( {1 - D} \right)^{2} }}\left| {I_{o} } \right|$$

The voltage and current stress are calculated for the two power diodes and switches:

10 $${ }V_{S1} = \frac{1}{1 - D}V_{in} ,{ }V_{S2} = \frac{1}{{\left( {1 - D} \right)^{2} }}V_{in}$$

11 $$I_{S1 - avg} = \frac{D}{{\left( {1 - D} \right)^{2} }}\left| {I_{o} } \right|,{ }I_{S2 - avg} = \frac{D}{1 - D}\left| {I_{o} } \right|$$

12 $${ }V_{D1} = \frac{1}{1 - D}V_{in} ,{ }V_{D2} = \frac{{2 - {\text{D}}}}{{\left( {1 - D} \right)^{2} }}V_{in}$$

13 $$I_{D1 - avg} = \frac{{\text{D}}}{1 - D}\left| {I_{o} } \right|,{ }I_{D2 - avg} = \left| {I_{o} } \right|$$

### Efficiency and power loss evaluation

**Figure 6:**
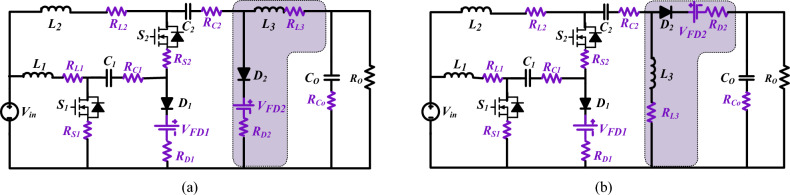
Equivalent circuit considering nonidealities. (**a**) Structure1; (**b**) Structure2.

Various parameters affect the power loss of the presented structures configuration, such as the diode's forward voltage drop and switching frequency, and component internal resistances. To obtain the number of losses in each circuit element, the circuit illustrated in Fig. 6 is used.

Switches and diodes have the following approximate *RMS* values of structures are:

14 $$\begin{gathered} I_{S1 - RMS} = \sqrt {\frac{{\int_{0}^{{DT_{S} }} {(i_{L1} + i_{L2} + i_{L3} )^{2} dt} }}{{T_{S} }}} = \frac{{\sqrt[{}]{D}}}{{\left( {1 - D} \right)^{2} }}\left| {I_{o} } \right| \hfill \\ I_{S2 - RMS} = \sqrt {\frac{{\int_{0}^{{DT_{S} }} {\left( {i_{L2} + i_{L3} } \right)^{2} dt} }}{{T_{S} }}} = \frac{\sqrt D }{{(1 - D)}}\left| {I_{o} } \right| \hfill \\ \end{gathered}$$

15 $$\begin{aligned} I_{D1 - RMS} & = \sqrt {\frac{{\int_{{DT_{S} }}^{{T_{S} }} {i_{L1}^{2} dt} }}{{T_{S} }}} = \frac{{DI_{o} }}{{\sqrt {(1 - D)^{3} } }} \\ I_{D2 - RMS} & = \sqrt {\frac{{\int_{{DT_{S} }}^{{T_{S} }} {\left( {i_{L2} + i_{L3} } \right)^{2} dt} }}{{T_{S} }}} = \frac{{I_{o} }}{{\sqrt {(1 - D)} }} \\ \end{aligned}$$

Moreover, to get the estimated *RMS* currents flowing through an inductor and capacitor, apply the following equations:

16 $$\begin{aligned} I_{L1 - RMS} & = \sqrt {\frac{{\int_{0}^{{T_{S} }} {i_{L1}^{2} dt} }}{{T_{S} }}} = \frac{D}{{\left( {1 - D} \right)^{2} }}\left| {I_{o} } \right| \\ I_{L2 - RMS} & = \sqrt {\frac{{\int_{0}^{{T_{S} }} {i_{L2}^{2} dt} }}{{T_{S} }}} = \frac{D}{{\left( {1 - D} \right)}}\left| {I_{o} } \right| \\ I_{L3 - RMS} & = \sqrt {\frac{{\int_{0}^{{T_{S} }} {i_{L3}^{2} dt} }}{{T_{S} }}} \approx \left| {I_{o} } \right| \\ \end{aligned}$$

17 $$\begin{aligned} I_{C1 - RMS} & = \sqrt {\frac{{\int_{0}^{{DT_{S} }} {(i_{L2} + i_{L3} )^{2} dt + \int_{{DT_{S} }}^{{T_{S} }} {i_{L1}^{2} dt} } }}{{T_{S} }}} = \frac{{D^{1/2} }}{{\left( {1 - D} \right)^{3/2} }}I_{o} \\ I_{C2 - RMS} & = \sqrt {\frac{D}{{\left( {1 - D} \right)}}} I_{o} ,I_{C3 - RMS} \approx I_{o} \\ \end{aligned}$$

In power switches, the total power loss (*P*_*S1,2-Total*_), is definite as the sum of leading power dissipations (*P*_*R-S*_) and the switching losses (*P*_*S-L*_). Assuming *R*_*S*_ is the power switch's conduction resistance, we can determine:

18 $$P^{S}_{Loss} = P_{S - Switching} + P_{S - Conduction}$$

19 $$\begin{aligned} P_{S - Conduction} & = R_{S1} \frac{D}{{\left( {1 - D} \right)^{4} }}\frac{{P_{o} }}{{R_{o} }} + R_{S2} \frac{D}{{\left( {1 - D} \right)^{2} }}\frac{{P_{o} }}{{R_{o} }} \\ P_{S - Switching} & = \frac{1}{2}I_{S1} V_{S1} t_{off1} f_{s} + \frac{1}{2}I_{S2} V_{S2} t_{off2} f_{s} + \frac{1}{2}C_{OSS} V_{S}^{2} f_{s} \\ & \quad \frac{1}{2}\frac{{P_{o} }}{{\left( {1 - D} \right)(2 - D)}}\left( {t_{off1} + t_{off2} } \right)f_{s} + \frac{1}{2}C_{OSS} V_{S}^{2} f_{s} \\ \end{aligned}$$

In addition, by using *V*_*FD*_ as its forward bias voltage and *R*_*FD*_ as its accelerative conduction resistance, the overall losses of the diodes can also be calculated as the sum of forward (*P*_*FD*_) and reverse bias losses (*P*_*FR*_*)*:

20 $$\begin{aligned} P^{D}_{Loss} & = P_{FD} + P_{FR} \\ P_{FD} & = R_{FD1} I_{D1 - RMS}^{2} + R_{FD2} I_{D2 - RMS}^{2} \\ P_{FR} & = V_{FD1} I_{D1} + V_{FD2} I_{D2} \\ \end{aligned}$$

Two types of losses in inductors: copper losses and core losses. Obtaining the core loss of inductors can be done as follows:

21 $$\begin{gathered} P_{L - Core} = P_{L - Core1} + P_{L - Core2} + P_{L - Core3} \hfill \\ P_{L - Core} = a_{1} B_{1}^{b} f_{1}^{c} l_{m1} A_{C1} + a_{2} B_{2}^{b} f_{2}^{c} l_{m2} A_{C2} + a_{3} B_{3}^{b} f_{3}^{c} l_{m3} A_{C3} \hfill \\ \end{gathered}$$

In this equation, *α, b*, and *c* parameters can be obtained from the data sheets. *f, B*, *l*_*m*_* ,* and* A*_*c*_ are the frequency, half of the ac flux, the core’s magnetic path length, and the area of the core, respectively. Considering *R*_*C*_ and *R*_*L*_, the following equation can be applied for determining equivalent series resistance (ESR) values of inductors, and capacitors; an inductor and capacitor's copper loss and total loss are defined as follows:

22 $$P_{L - Copper} = R_{L1} I_{L1 - RMS}^{2} + R_{L2} I_{L2 - RMS}^{2} + R_{L3} I_{L3 - RMS}^{2}$$

23 $$P^{L}_{Loss} = P_{L - Core} + P_{L - Copper}$$

Using *R*_*C*_ to determine equivalent series resistances (ESRs) and power losses for capacitors, the following equation can be used:

24 $$P_{C - Total} = \mathop \sum \limits_{i = 1}^{i = 3} \left( {P_{Ci} } \right) = \mathop \sum \limits_{i = 1}^{i = 3} \left( {R_{Ci} I_{Ci - RMS}^{2} } \right)$$

Accordingly, the sum of the power losses resulting from diodes, inductors, capacitors, and power switches constitutes the overall power losses of the suggested converter.

25 $$P_{Loss - Total} = P^{S}_{Loss} + P^{D}_{Loss} + P^{L}_{Loss} + P^{C}_{Loss}$$

Finally, the efficiency of presented structures:

26 $$\eta = \frac{{P_{o} }}{{P_{Loss - Total} + P_{o} }} = \frac{1}{{\frac{{P_{Loss - Total} }}{{R_{o} I_{o}^{2} }} + 1}}$$

**Figure 7:**
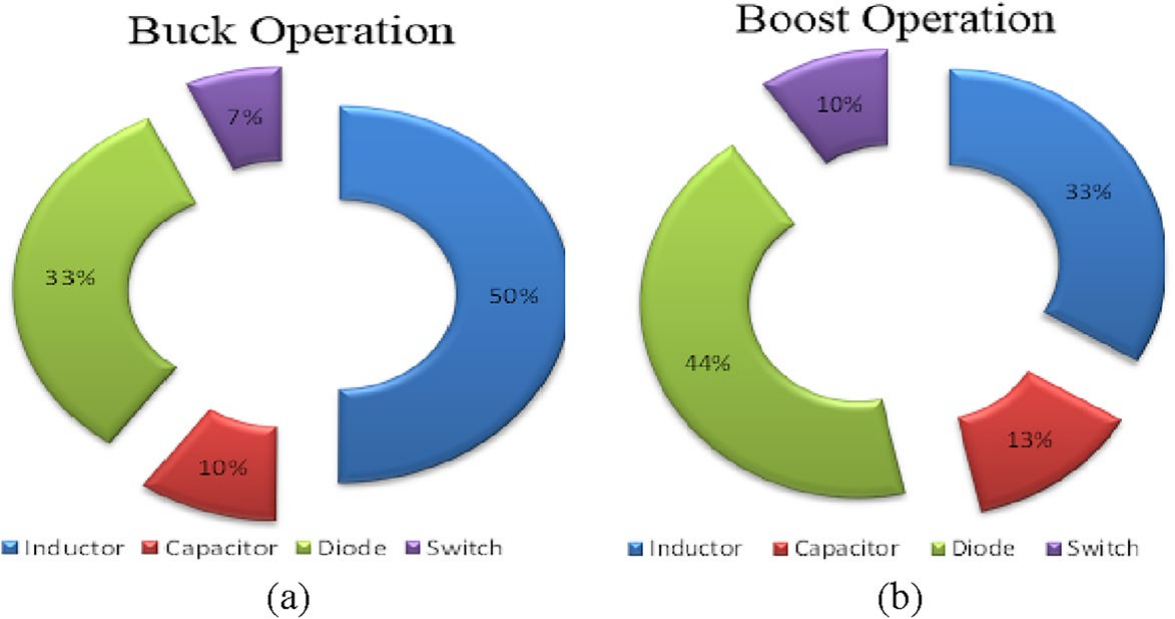
Power loss in different components in the presented structures. (**a**) Buck operation, (**b**) Boost operation.

Figure 7 shows the charts in Buck**/**Boost operation to understand the power losses of each element portion. In the buck operation, the efficiency is determined as less than that in the boost operation. The converter possesses a higher input current compared to the introduced converter output current in boost mode. However, when the step-down mode is on, the output current is higher than the input current. Therefore, compared to the step-up mode, there is a decrease in efficiency in the step-down mode. The power losses of *S*, *D*, *L* and *C* are determined as (18), (20), (23) and (24) in buck/boost mode.

### Effect of parasitic parameters on the circuit

The simplified proposed circuit includes parasitic parameters for structure1, such as parasitic parameters of the inductors (*L*_*3*_*, L*_*2*_, and* L*_*1*_), the capacitors (*C*_*2*_ and* C*_*1*_), the diodes (*D*_*2*_ and* D*_*1*_), and the MOSFETs (*S*_*2*_ and *S*_*1*_), which is represented in Fig. 6. *V*_*FD1*_ and *V*_*FD2*_ represent threshold voltages of diodes. Therefore, the equations for inductor voltages involving parasitic parameters (*R*_*L3*_*, R*_*L2*_*, R*_*L1*_*, R*_*C2*_*, R*_*C1,*_* R*_*D2*_,* R*_*D1*_*, R*_*S2*_*, R*_*S1*_) can be calculated as follows:

27 $$State1:\left\{ \begin{gathered} V_{L1} = V_{in} - R_{L1} I_{L1} - R_{S1} I_{S1} \hfill \\ = V_{in} - R_{L1} \frac{{D^{2} \left( {2 - D} \right)}}{{\left( {1 - D} \right)^{4} }}\frac{{V_{in} }}{{R_{O} }} - R_{S1} \frac{{D^{2} \left( {2 - D} \right)}}{{\left( {1 - D} \right)^{4} }}\frac{{V_{in} }}{{R_{O} }} \hfill \\ V_{L2} = V_{in} - R_{L2} I_{L2} - R_{S1} I_{S1} - R_{S2} I_{S2} - R_{C1} I_{C1} + V_{C1} \hfill \\ = V_{in} - R_{L2} \frac{{D^{2} \left( {2 - D} \right)}}{{\left( {1 - D} \right)^{3} }}\frac{{V_{in} }}{{R_{O} }} - R_{S1} \frac{{D^{2} \left( {2 - D} \right)}}{{\left( {1 - D} \right)^{4} }}\frac{{V_{in} }}{{R_{O} }} - R_{S2} \frac{{D^{2} \left( {2 - D} \right)}}{{\left( {1 - D} \right)^{3} }}\frac{{V_{in} }}{{R_{O} }} - R_{C1} \frac{{D^{2} \left( {2 - D} \right)}}{{\left( {1 - D} \right)^{3} }}\frac{{V_{in} }}{{R_{O} }} + V_{C1} \hfill \\ V_{L3} = - R_{L3} I_{L3} - R_{S1} I_{S1} - R_{S2} I_{S2} - R_{C1} I_{C1} - R_{C2} I_{C2} + V_{C1} + V_{C2} - V_{O} \hfill \\ = - R_{L3} \frac{{D\left( {2 - D} \right)}}{{\left( {1 - D} \right)^{2} }}\frac{{V_{in} }}{{R_{O} }} - R_{S1} \frac{{D^{2} \left( {2 - D} \right)}}{{\left( {1 - D} \right)^{4} }}\frac{{V_{in} }}{{R_{O} }} - R_{S2} \frac{{D^{2} \left( {2 - D} \right)}}{{\left( {1 - D} \right)^{3} }}\frac{{V_{in} }}{{R_{O} }} - R_{C1} \frac{{D^{2} \left( {2 - D} \right)}}{{\left( {1 - D} \right)^{3} }}\frac{{V_{in} }}{{R_{O} }} - R_{C2} \frac{{{\text{D}}\left( {2 - D} \right)}}{{\left( {1 - D} \right)^{2} }}\frac{{V_{in} }}{{R_{O} }} + V_{C1} + V_{C2} - V_{O} \hfill \\ \end{gathered} \right.$$

28 $$State 2: \left\{ \begin{gathered} V_{L1} = V_{in} - R_{L1} I_{L1} - R_{D1} I_{D1} - R_{C1} I_{C1} - V_{C1} - V_{FD1} \hfill \\ = V_{in} - R_{L1} \frac{{D^{2} \left( {2 - D} \right)}}{{\left( {1 - D} \right)^{4} }}\frac{{V_{in} }}{{R_{o} }} - R_{D1} \frac{{D^{2} \left( {2 - D} \right)}}{{\left( {1 - D} \right)^{3} }}\frac{{V_{in} }}{{R_{o} }} - R_{C1} \frac{{D^{2} \left( {2 - D} \right)}}{{\left( {1 - D} \right)^{4} }}\frac{{V_{in} }}{{R_{o} }} \hfill \\ - V_{C1} - V_{FD1} \hfill \\ V_{L2} = V_{in} - R_{L2} I_{L2} - R_{D2} I_{D2} - R_{C2} I_{C2} - V_{C2} - V_{FD2} \hfill \\ = V_{in} - R_{L2} \frac{{D^{2} \left( {2 - D} \right)}}{{\left( {1 - D} \right)^{3} }}\frac{{V_{in} }}{{R_{o} }} + R_{D2} \frac{{D\left( {2 - D} \right)^{2} }}{{\left( {1 - D} \right)^{4} }}\frac{{V_{in} }}{{R_{o} }} - R_{C2} \frac{{D^{2} \left( {2 - D} \right)}}{{\left( {1 - D} \right)^{3} }}\frac{{V_{in} }}{{R_{o} }} \hfill \\ - V_{C2} - V_{FD2} \hfill \\ = - R_{L3} \frac{{D\left( {2 - D} \right)}}{{\left( {1 - D} \right)^{2} }}\frac{{V_{in} }}{{R_{o} }} + R_{D2} \frac{{D\left( {2 - D} \right)^{2} }}{{\left( {1 - D} \right)^{4} }}\frac{{V_{in} }}{{R_{o} }} \hfill \\ V_{L3} = - R_{L3} I_{L3} - R_{D2} I_{D2} - V_{FD2} - V_{0} \hfill \\ - V_{FD2} - V_{0} \hfill \\ \end{gathered} \right.$$

By applying the inductors' voltage-second balancing concept, the average voltages of capacitors *C*_*1*_ and *C*_*2*_ and the voltage gain ratio involving parasitic parameters can be calculated by:

29 $$V_{C1} = \frac{{\begin{array}{*{20}c} { (1 - D)^{4} R_{o} V_{in} - D^{2} \left( {2 - D} \right)R_{L1} V_{in} - D^{3} \left( {2 - D} \right)V_{in - } } \\ { - D^{2} \left( {2 - D} \right)\left( {1 - D} \right)R_{D1} V_{in} - D^{2} \left( {2 - D} \right)\left( {1 - D} \right)R_{C1} V_{in} - \left( {1 - D} \right)^{5} R_{o} V_{FD1} } \\ \end{array} }}{{(1 - D)^{5} R_{o} }}$$

30 $$V_{C2} = \frac{{\begin{array}{*{20}c} {\left( {1 - D} \right)^{4} R_{o} V_{in} - D^{3} \left( {2 - D} \right)R_{L1} V_{in} + } \\ {D^{2} \left( {2 - D} \right)\left( {1 - D} \right)^{2} R_{L2} V_{in} - D^{3} \left( {2 - D} \right)R_{S1} V_{in} - } \\ {D^{3} \left( {2 - D} \right)\left( {1 - D} \right)^{2} R_{S2} V_{in} - D^{3} \left( {2 - D} \right)R_{D1} V_{in} } \\ { - D\left( {2 - D} \right)\left( {1 - D} \right)^{2} R_{D2} V_{in} - D\left( {1 - D} \right)^{5} R_{o} V_{FD1} + } \\ {\left( {1 - D} \right)^{6} R_{o} V_{FD2} } \\ \end{array} }}{{(1 - D)^{6} R_{o} }}$$

31 $$M = \frac{{V_{o} }}{{V_{in} }} = \frac{\begin{gathered} D\left( {2 - D} \right)\left( {1 - D} \right)^{4} R_{o} - D^{3} \left( {2 - D} \right)R_{L1} - D^{3} \left( {2 - D} \right)\left( {1 - D} \right)^{2} R_{L2} - D\left( {2 - D} \right)\left( {1 - D} \right)^{4} R_{L3} + D^{3} \left( {2 - D} \right)R_{S1} \hfill \\ - D^{3} \left( {2 - D} \right)\left( {1 - D} \right)^{2} R_{S2} - D^{3} \left( {2 - D} \right)\left( {1 - D} \right)R_{D1} - D\left( {2 - D} \right)\left( {1 - D} \right)^{4} R_{D2} \hfill \\ - D^{3} \left( {2 - D} \right)^{2} \left( {1 - D} \right)R_{C1} - D^{2} \left( {2 - D} \right)^{2} \left( {1 - D} \right)^{3} R_{C2} - D\left( {1 - D} \right)^{5} R_{o} \frac{{V_{FD1} }}{{V_{in} }} - \left( {1 - D} \right)^{6} R_{o} \frac{{V_{FD2} }}{{V_{in} }} \hfill \\ \end{gathered} }{{(1 - D)^{6} R_{o} }}$$

It is possible to attain the average output voltage of an inductor by as the parasitic elements based on the volt-second balance principle:

32 $$\begin{gathered} V_{o} = \frac{{D\left( {2 - D} \right)\left( {1 - D} \right)^{4} V_{in} - D\left( {1 - D} \right)^{5} V_{FD1} - \left( {1 - D} \right)^{6} V_{FD2} }}{{\left( {1 - D} \right)^{6} + \alpha_{1} \frac{{R_{L1} }}{{R_{o} }} + \alpha_{2} \frac{{R_{L2} }}{{R_{o} }} + \alpha_{3} \frac{{R_{L3} }}{{R_{o} }} + \alpha_{4} \frac{{R_{S1} }}{{R_{o} }} + + \alpha_{5} \frac{{R_{S2} }}{{R_{o} }} + \alpha_{6} \frac{{R_{D1} }}{{R_{o} }} + \alpha_{7} \frac{{R_{D2} }}{{R_{o} }} + + \alpha_{8} \frac{{R_{C1} }}{{R_{o} }} + \alpha_{9} \frac{{R_{C2} }}{{R_{o} }}}} \hfill \\ \alpha_{1} = D^{2} \left( {1 - D} \right)^{2} ,\alpha_{2} = D^{2} \left( {1 - D} \right)^{4} ,\alpha_{3} = \left( {1 - D} \right)^{3} ,\alpha_{4} = D^{2} \left( {1 - D} \right)^{2} ,\alpha_{5} = D^{2} \left( {1 - D} \right)^{4} , \hfill \\ \alpha_{6} = D^{2} \left( {1 - D} \right)^{3} ,\alpha_{7} = \left( {1 - D} \right)^{6} ,\alpha_{8} = D\left( {2 - D} \right)\left( {1 - D} \right)^{3} ,\alpha_{9} = D\left( {2 - D} \right)\left( {1 - D} \right)^{5} \hfill \\ \end{gathered}$$

**Figure 8:**
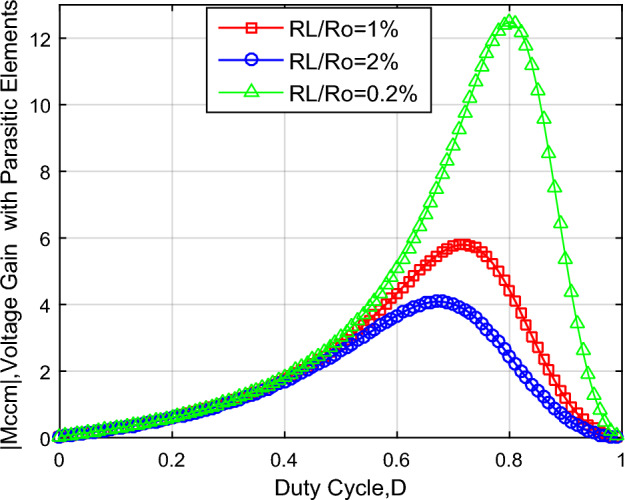
|M_CCM_| considering the parasitic elements.

The voltage conversion ratio |M_CCM_*|* value versus parasitic resistance *R*_*L*_ and *V*_*FD*_ is presented in Fig. 8 and the |M_CCM_*|* is impacted by parasitic elements. As a result, *V*_*in*_ = *20V*, *V*_*FD1,2*_ = *0.9V*, and* R*_*L1,2,3*_ = *β* × *R*_*o*_, where *β* = *1%, 2%, 0.2%*.

## Analysis and design parameters

### Determination of the amount of ripple inductors

Ripples in inductor currents *i*_*L*1,_* i*_*L*2_ and *i*_*L*3_ are obtained in Figs. 4a and 5a:

33 $$\Delta i_{L1,2,3} = \frac{{DV_{L1,2,3} }}{{L_{1,2,3} f_{s} }}$$

To strategy the inductors in the proposed converter, the current ripples are measured. Regarding the peak-to-peak current (Figs. 4a, 5a) and supposing that current ripples in inductors of (33), the needed values of inductances and ripples inductor currents are:

34 $$L_{1,2,3} = \frac{{DV_{L} }}{{\alpha \% \Delta i_{L} f_{s} }}, \Delta i_{L} \le \alpha \% i_{L} \alpha \le 30\%$$

Substituting *I*_*L1,2,3*_ from (9) to (34), the following relation can be obtained:

35 $$L_{1} \ge \frac{{\left( {1 - D} \right)^{4} }}{{\alpha \% D(2 - D)^{2} }} \times \frac{{R_{o} }}{{f_{s} }},L_{2} \ge \frac{{\left( {1 - D} \right)^{2} }}{\alpha \% D} \times \frac{{R_{o} }}{{f_{s} }},L_{3} \ge \frac{1 - D}{{\alpha \% D}} \times \frac{{R_{o} }}{{f_{s} }}$$

For solar panels to extract more power and for fuel cells and batteries to last longer, the input current ripple needs to be suppressed to a rational value^49,50^. Moreover, an inductor's inductance size determines its current ripple limit. Thus, taking into account the maximum satisfactory current ripple for each inductor, the output/input current ripple magnitudes can be determined for structure1and 2 as follows:

36 $$\left\{ {\begin{array}{*{20}l} {\Delta i_{in} = \Delta i_{1} + \Delta i_{2} = \frac{{V_{O} (1 - D)^{2} }}{{L_{1} f_{s} (2 - D)}} + \frac{{V_{O} (1 - D)}}{{L_{2} f_{s} }}, } \hfill \\ {L_{1} = 152\mu H,\;L_{1} = 810\mu H} \hfill \\ {\Delta i_{L3} = \frac{{V_{o} \left( {1 - D} \right)}}{{L_{3} f_{s} D}},\;L_{3} = 1.7mH} \hfill \\ \end{array} } \right.$$

**Figure 9:**
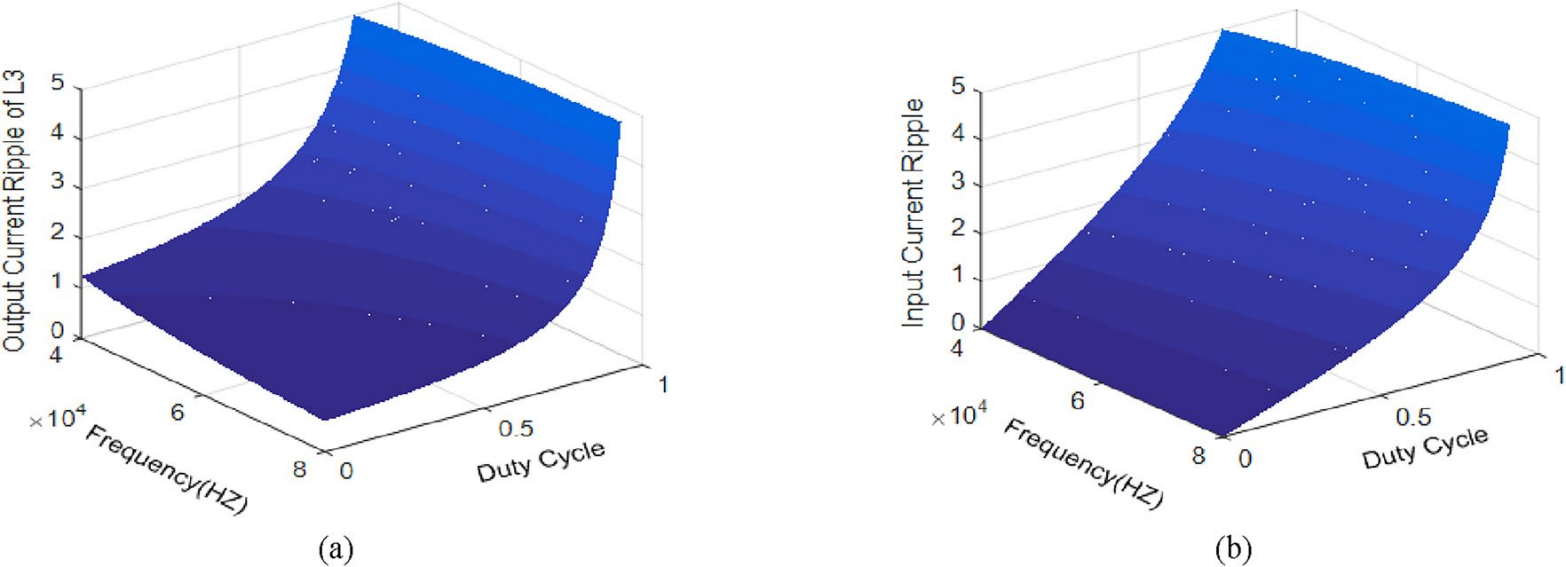
Current ripple plot versus *fs* and *D*. (**a**) Output current; (**b**) Input current.

The output and input current ripple of *L*_*3*_ and *L*_*1*_ variation against duty cycle and switching frequency is shown in Fig. 9.

### Drop in voltage of inductor

Across each inductor, a net voltage drop exists caused by the inductor’s internal resistance. Based on (37) it should be the percentage of the average voltage drop across each inductor based on the input:

37 $$\begin{array}{*{20}c} {\Delta V_{L1} = \frac{{I_{L1} \times R_{L1} }}{{V_{in} }} \times 100\% = \frac{{D^{2} (2 - D)^{2} }}{{(1 - D)^{4} }}\frac{{R_{L1} }}{{R_{o} }}} \\ {\Delta V_{L2} = \frac{{I_{L2} \times R_{L2} }}{{V_{in} }} \times 100\% = \frac{{D^{3} \left( {2 - D} \right)}}{{(1 - D)^{3} }}\frac{{R_{L2} }}{{R_{o} }}} \\ {\Delta V_{L3} = \frac{{I_{L3} \times R_{L3} }}{{V_{in} }} \times 100\% = \frac{{D\left( {2 - D} \right)}}{{(1 - D)^{2} }}\frac{{R_{L3} }}{{R_{o} }}} \\ \end{array}$$

**Figure 10:**
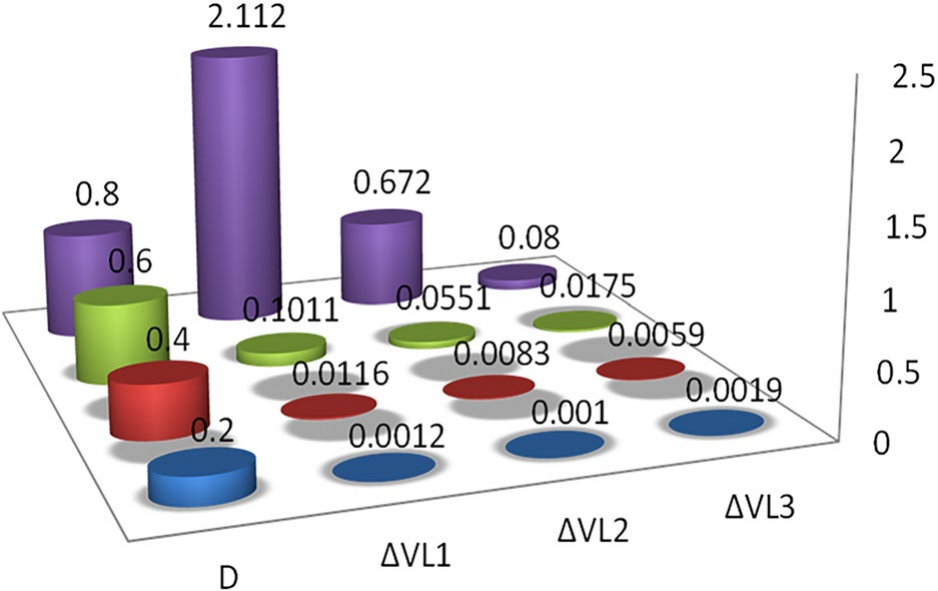
Voltage drop across *L*_*1*_*, L*_*2*_ and *L*_*3*_ inductors as a percentage of the output voltage.

Across each inductor, the voltage drop as the output voltage percentage is according to the internal resistance, load resistance, and duty ratio. For various duty cycles, Fig. 10 shows the voltage drop through individually inductor as a percentage of the output voltage for different Duty cycle (0.2, 0.4, 0.6, and 0.8). Voltage drops across the inductor are similar at low-duty cycles, while at high-duty cycles, voltage drops increase with the source. The reason is that the input current is very high at a higher duty cycle value.

### Operation of proposed converter in DCM

**Figure 11:**
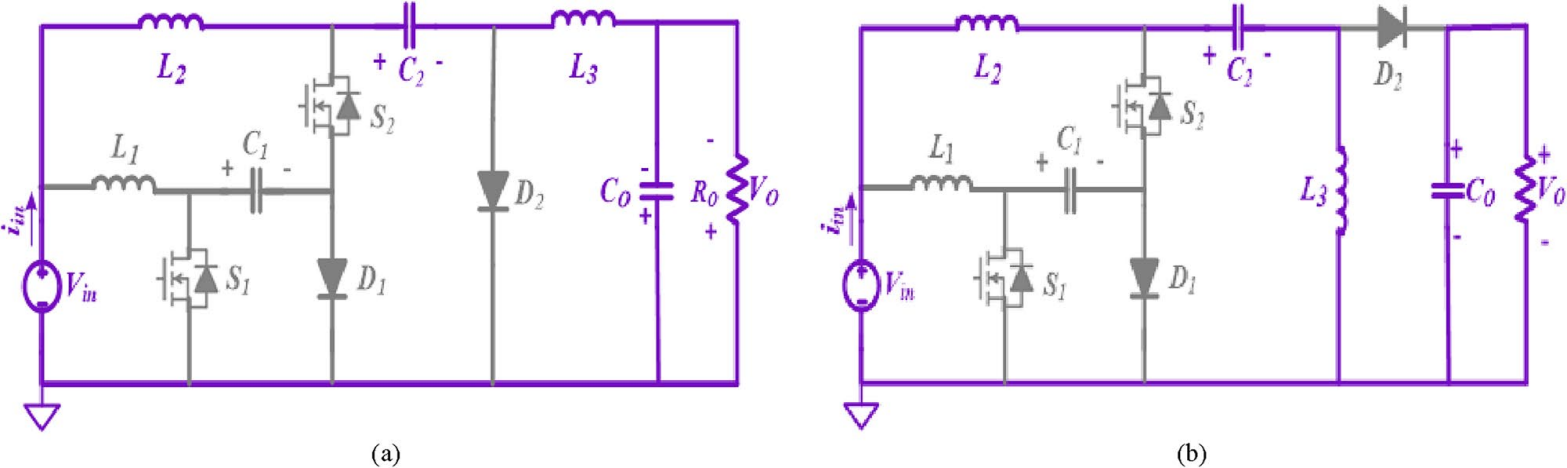
State 3 operation in DCM. (**a**)Structure 1; (**b**) Structure2.

**Figure 12:**
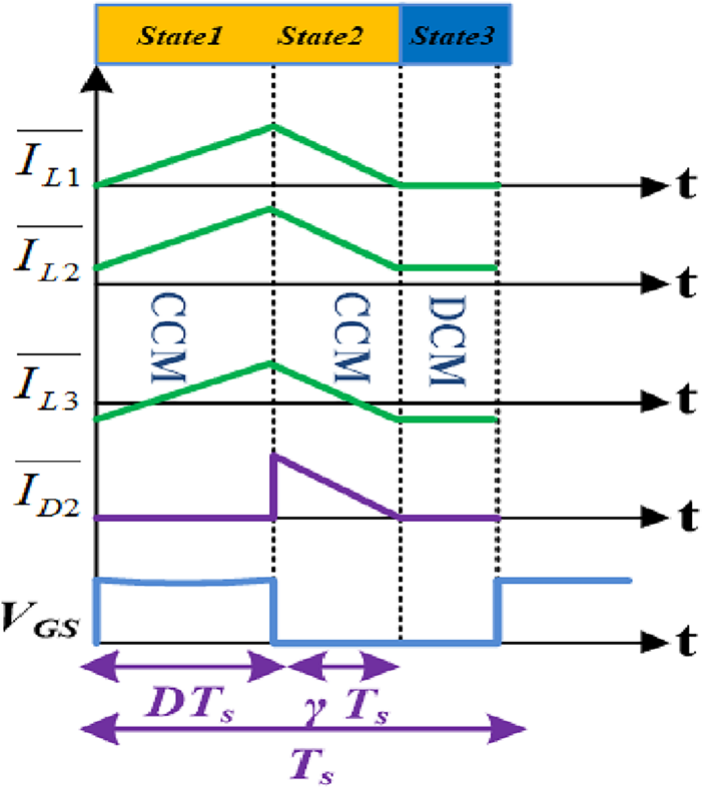
Switching waveform in DCM.

The suggested converter works in Discontinuous conduction mode (DCM), So that current of the diode *D*_*2*_ comes to zero some time before the begin of the another exchanging cycle. The DCM configuration plot and key waveforms of the suggested converter for DCM operation are portrayed in Figs. 11 and 12, respectively. The operation of the proposed converter can be partitioned into third states. The states 1 and 2 are equal to the CCM operation and the state3 (DCM operation), all the switches *S*_*1*_, *S*_*2*_ and diodes *D*_*1*_*, D*_*2*_ are turn off.

By using the current and voltage Eqs. (3)–(4) in DCM, the voltage gain is obtained as,

38 $$\left( {\frac{{V_{o} }}{{V_{in} }}} \right)_{DCM} = \frac{{D\left( {D + 2\gamma } \right)}}{{\gamma \left( {1 - D} \right)}}$$

Again, by utilizing the principle of current-second balance to the capacitor *C*_*o*_, the following equation is derived.

39 $$I_{o} = \frac{\gamma }{2}\Delta I_{L3}$$

By using (38) and (39), $\gamma$ is calculated as,

40 $$\gamma = \frac{{2\tau_{L3} + \sqrt {4\tau_{L3}^{2} + 2\tau_{L3} \left( {2 - D} \right)} }}{2 - D}$$

From (38) and (40), the voltage gain in DCM is obtained as,

41 $$\left( {\frac{{V_{o} }}{{V_{in} }}} \right)_{DCM} = \frac{1}{1 - D} \times \left[ {\frac{{D\left( {2 - D} \right) + 4\tau_{L3} + 2\left( {4\tau_{L3}^{2} + 2\tau_{L3} \left( {2 - D} \right)} \right)^{\frac{1}{2}} }}{{2\tau_{L3} + \left( {4\tau_{L3}^{2} + 2\tau_{L3} \left( {2 - D} \right)} \right)^{\frac{1}{2}} }}} \right]$$

where the inductor (${L}_{3}$) time constant $({\tau }_{L})$ is $L3{f}_{S}/ {R}_{o}$*.*

Due to the dependence of the DCM converter's voltage gain on its parameters and the high input current ripple, DCM operation is typically not advised.

### Determination of boundary condition

Boundary conditions of the proposed converter can be obtained by equating M_CCM_ = M_DCM_. It is essential to design the discrete inductors that must guarantee the continuous conduction mechanism for the presented converter. Taking into account the aforementioned necessity, the condition of CCM is determined in terms of the alternative circuit (Figs. 4a, 5a) for inductors *L*_*1*_ and *L*_*2,3*_, using the following:

42 $$\left\{ {\begin{array}{*{20}l} {L_{1} \ge \frac{{\left( {1 - D} \right)^{4} }}{2 - D} \times \frac{{R_{o} }}{{f_{s} }}} \hfill \\ {L_{2} \ge \frac{{\left( {1 - D} \right)^{2} }}{D} \times \frac{{R_{o} }}{{f_{s} }}} \hfill \\ {L_{3} \ge \frac{{\left( {1 - D} \right)^{2} }}{1} \times \frac{{R_{o} }}{{f_{s} }}} \hfill \\ \end{array} { }} \right.$$

**Figure 13:**
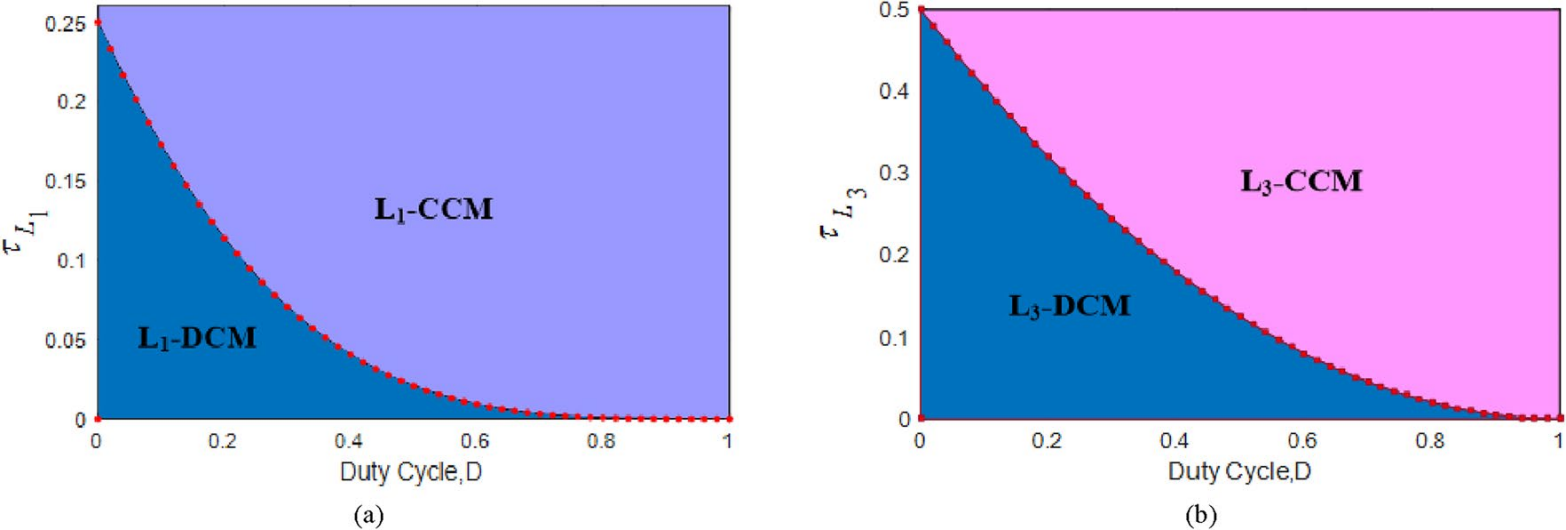
Plot of boundary normalized inductor time constant versus *D*. (**a**) Inductor *L*_*1*_; (**b**) Inductor *L*_*3*._

The *τ*_*LB*_ (boundary condition*)* for *L*_*1*,_* L*_*2*_*,* and *L*_*3*_ are as follows. Moreover, the area over each curve of Fig. 13 is the continuous conduction mode and the area below represents the discontinuous conduction mode, which are as follows:

43 $$\left\{ {\begin{array}{*{20}c} {\tau_{L1B} = {\raise0.7ex\hbox{${\left( {1 - D} \right)^{4} }$} \!\mathord{\left/ {\vphantom {{\left( {1 - D} \right)^{4} } {2\left( {2 - D} \right)}}}\right.\kern-0pt} \!\lower0.7ex\hbox{${2\left( {2 - D} \right)}$}}} \\ {\tau_{L2B} = {\raise0.7ex\hbox{${\left( {1 - D} \right)^{2} }$} \!\mathord{\left/ {\vphantom {{\left( {1 - D} \right)^{2} } {2D}}}\right.\kern-0pt} \!\lower0.7ex\hbox{${2D}$}}} \\ {\tau_{L3B} = {\raise0.7ex\hbox{${\left( {1 - D} \right)^{2} }$} \!\mathord{\left/ {\vphantom {{\left( {1 - D} \right)^{2} } 2}}\right.\kern-0pt} \!\lower0.7ex\hbox{$2$}}} \\ \end{array} { }} \right.$$

### Determination of the amount of ripple capacitors

Voltage ripples through the capacitors *C*_*o*_, *C*_2_ and *C*_1_ are:

44 $$\Delta V_{Co} = \frac{{Di_{Co} }}{{C_{o} f_{s} }},\,\Delta V_{C1,2} = \frac{{Di_{C1,2} }}{{C_{1,2} f_{s} }}$$

Also, based on the following conditions, the capacitances of each capacitor can be obtained as:

45 $$\left\{ {\begin{array}{*{20}l} {\Delta v_{C1,2} \le \beta \% V_{C1,2} } \hfill & {\beta \le 2\% } \hfill \\ {\Delta v_{Co} \le \delta \% V_{o} } \hfill & {\delta \le 0.5\% } \hfill \\ \end{array} } \right.$$

46 $$\left\{ {\begin{array}{*{20}l} {C_{1} = \frac{{D^{2} \left( {2 - D} \right)R_{o} }}{{\beta \% \left( {1 - D} \right)^{2} f_{s} }}} \hfill \\ {C_{2} = \frac{{D^{2} \left( {2 - D} \right)R_{o} }}{{\beta \% f_{s} }} \quad \left( {structure1} \right)} \hfill \\ {C_{2} = \frac{{D^{2} \left( {2 - D} \right)R_{o} }}{{\beta \% \left( {1 - D} \right)^{2} f_{s} }}\quad \left( {structure1} \right)} \hfill \\ {C_{o} = \frac{{\left( {1 - D} \right)^{2} R_{o} }}{{\beta \% \left( {2 - D} \right)f_{s} }}} \hfill \\ \end{array} } \right.$$

It is necessary to decrease the voltage ripple of the output capacitor to a reasonable level in order to stabilize the transferred power to the next part of the circuit and extend the lifespan of the components. Moreover, the voltage ripple limit of a capacitor is based on its capacitance size. The voltage ripple of *C*_*1*_ and the output capacitor voltage ripple are achieved as follows:

47 $$\left\{ {\begin{array}{*{20}c} {\Delta v_{C1} = \frac{{DI_{C1} }}{{C_{1} f_{s} }},C_{1} = 33\mu F} \\ {\Delta v_{Co} = \frac{{DI_{C0} }}{{C_{o} f_{s} }},C_{o} = 64\mu F} \\ \end{array} } \right.$$

**Figure 14:**
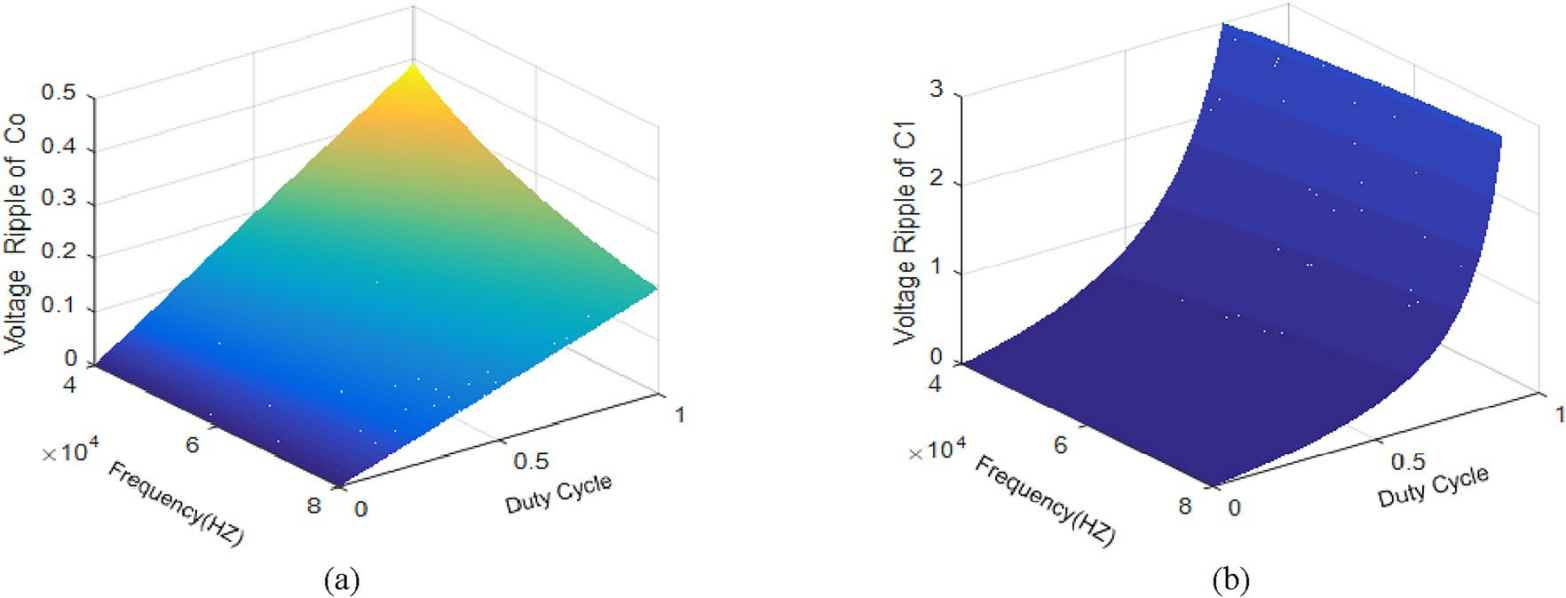
Voltage ripple plot versus fs and D. (**a**) *C*_*o*_; (**b**) *C*_*1*_*.*

The voltage ripple of *C*_o_ and *C*_*1*_ variation against the duty cycle and switching frequency is shown in Fig. 14.

### Calculation of duty cycle

It is possible to calculate the duty cycle in order to determine the desired output voltage given an input voltage. Based on (6) is obtained:

48 $$D = \sqrt {V_{in}^{2} + V_{in} V_{o} } /\left( {V_{o} + V_{in} } \right) + 1$$

## Small signal modeling

The equations of the dual states from Fig. 4a and b (structure1), can be calculated by:

49 $$State1:\left\{ {\begin{array}{*{20}l} {L_{1} \frac{{di_{L1} }}{dt} = v_{in} } \hfill \\ {L_{2} \frac{{di_{L2} }}{dt} = v_{in} + v_{C1} } \hfill \\ {L_{3} \frac{{di_{L3} }}{dt} = v_{C1} - v_{C2} - v_{Co} } \hfill \\ {C_{1} \frac{{dv_{C1} }}{dt} = - i_{L3} - i_{L2} } \hfill \\ {C_{2} \frac{{dv_{C2} }}{dt} = - i_{L3} } \hfill \\ {C_{o} \frac{{dv_{Co} }}{dt} = - i_{L3} - \frac{{v_{Co} }}{{R_{o} }}} \hfill \\ \end{array} } \right.\,State 2:\left\{ {\begin{array}{*{20}l} {L_{1} \frac{{di_{L1} }}{dt} = v_{in} - v_{C1} } \hfill \\ {L_{2} \frac{{di_{L2} }}{dt} = v_{in} - v_{C2} } \hfill \\ {L_{3} \frac{{di_{L3} }}{dt} = - v_{Co} } \hfill \\ {C_{1} \frac{{dv_{C1} }}{dt} = i_{L1} } \hfill \\ {C_{2} \frac{{dv_{C2} }}{dt} = i_{L2} } \hfill \\ {C_{o} \frac{{dv_{Co} }}{dt} = - i_{L3} - \frac{{v_{Co} }}{{R_{o} }}} \hfill \\ \end{array} } \right.$$

This state-average model^51^ can be simply obtained using the averaging method and Eq. (49):

50 $$State1\left\{ {\begin{array}{*{20}c} {L_{1} \frac{{d\left\langle {i_{L1} } \right\rangle }}{dt} = d\left( {\left\langle {v_{in} } \right\rangle } \right) + \left( {1 - d} \right)\left( {v_{in} - \left\langle {v_{C1} } \right\rangle } \right)} \\ {L_{2} \frac{{d\left\langle {i_{L1} } \right\rangle }}{dt} = d\left( {v_{in} + \left\langle {v_{C1} } \right\rangle } \right) - \left( {1 - d} \right)\left( {v_{in} - \left\langle {v_{C2} } \right\rangle } \right)} \\ {L_{3} \frac{{d\left\langle {i_{L1} } \right\rangle }}{dt} = - d\left( {v_{C1} + v_{C2} - \left\langle {v_{Co} } \right\rangle } \right) - \left( {1 - d} \right)\left( {\left\langle {v_{Co} } \right\rangle } \right)} \\ \end{array} } \right.$$

51 $$State2\left\{ {\begin{array}{*{20}c} {C_{1} \frac{{d\left\langle {v_{C1} } \right\rangle }}{dt} = d\left( { - i_{L3} - \left\langle {i_{L2} } \right\rangle } \right) - \left( {1 - d} \right)\left\langle {i_{L1} } \right\rangle } \\ {C_{2} \frac{{d\left\langle {v_{C2} } \right\rangle }}{dt} = d\left( { - \left\langle {i_{L3} } \right\rangle } \right) + \left( {1 - d} \right)\left( {\left\langle {i_{L2} } \right\rangle } \right)} \\ {C_{o} \frac{{d\left\langle {v_{Co} } \right\rangle }}{dt} = - d\left( {\left\langle {i_{L3} } \right\rangle + \left\langle {\frac{{v_{Co} }}{{R_{o} }}} \right\rangle } \right) - \left( {1 - d} \right)\left( {\left\langle {i_{L3} } \right\rangle + \left\langle {\frac{{v_{Co} }}{{R_{o} }}} \right\rangle } \right)} \\ \end{array} } \right.$$

Also, the average values of *i*_*L3*_*, i*_*L2*_*, i*_*L1*_*, v*_*Co*_, *v*_*C2*_*, v*_*C1*_ and *v*_*in*_ are $\langle {i}_{L3}\rangle , \langle {i}_{L2}\rangle ,\langle {i}_{L1}\rangle ,\langle {v}_{C0}\rangle ,\langle {v}_{C2}\rangle , \langle {v}_{C1}\rangle$ and $\langle {v}_{in}\rangle$ respectively. In order to derive the small signal model, the small AC values of the elements mentioned above must be determined as follows:$\widehat{{i}_{L3}}, \widehat{{i}_{L2}},$ $\widehat{{i}_{L1}}$, $\widehat{{v}_{c0}}$,$\widehat{{v}_{c2}}$, $\widehat{{v}_{c1}}$, $\widehat{{v}_{in}}$ and $\widehat{d}$. Besides, the associations among mean, AC, and DC values can be obtained as follows:

52 $$\left\{ {\begin{array}{*{20}l} {\left\langle {v_{in} } \right\rangle = V_{in} + \mathop {v_{in} }\limits^{ \wedge } } \hfill \\ {\begin{array}{*{20}c} {\left\langle {v_{C1} } \right\rangle = V_{C1} + \mathop {v_{C1} }\limits^{ \wedge } } \\ {\left\langle {v_{C2} } \right\rangle = V_{C2} + \mathop {v_{C2} }\limits^{ \wedge } } \\ \end{array} } \hfill \\ {\left\langle {v_{Co} } \right\rangle = V_{Co} + \mathop {v_{Co} }\limits^{ \wedge } } \hfill \\ {\left\langle {i_{L1} } \right\rangle = I_{L1} + \mathop {i_{L1} }\limits^{ \wedge } } \hfill \\ {\begin{array}{*{20}c} {\left\langle {i_{L2} } \right\rangle = I_{L2} + \mathop {i_{L2} }\limits^{ \wedge } } \\ {\left\langle {i_{L3} } \right\rangle = I_{L3} + \mathop {i_{L3} }\limits^{ \wedge } } \\ \end{array} } \hfill \\ {\left\langle d \right\rangle = D + \mathop d\limits^{ \wedge } } \hfill \\ \end{array} } \right.,\left\{ {\begin{array}{*{20}l} {\left| {\mathop {v_{in} }\limits^{ \wedge } } \right| \ll \left| {V_{in} } \right|} \hfill \\ {\begin{array}{*{20}c} {\left| {\mathop {v_{C1} }\limits^{ \wedge } } \right| \ll \left| {V_{C1} } \right|} \\ {\left| {\mathop {v_{C2} }\limits^{ \wedge } } \right| \ll \left| {V_{C2} } \right|} \\ \end{array} } \hfill \\ {\left| {\mathop {v_{Co} }\limits^{ \wedge } } \right| \ll \left| {V_{Co} } \right|} \hfill \\ {\left| {\mathop {i_{L1} }\limits^{ \wedge } } \right| \ll \left| {I_{L1} } \right|} \hfill \\ {\begin{array}{*{20}c} {\left| {\mathop {i_{L2} }\limits^{ \wedge } } \right| \ll \left| {I_{L2} } \right|} \\ {\left| {\mathop {i_{L3} }\limits^{ \wedge } } \right| \ll \left| {I_{L3} } \right|} \\ \end{array} } \hfill \\ {\left| {\mathop d\limits^{ \wedge } } \right| \ll \left| D \right|} \hfill \\ \end{array} } \right.$$

To obtain AC and DC values and remove the high-order small signal terms, we substitute (52) into (51) and (50):

53 $$y = Cx + Eu, K\dot{x} = Ax + Bu$$

With

54 $$x = \left[ {\begin{array}{*{20}c} {\widehat{{i_{L1} }}} & {\begin{array}{*{20}c} {\widehat{{i_{L2} }}} & {\begin{array}{*{20}c} {\widehat{{i_{L3} }}} & {\widehat{{v_{C1} }}} & {\widehat{{v_{C2} }}} \\ \end{array} } & {\widehat{{v_{Co} }}} \\ \end{array} } \\ \end{array} } \right]^{T} ,\; u = \left[ {\begin{array}{*{20}c} {\hat{d}} & {\widehat{{v_{in} }}} \\ \end{array} } \right]^{T}$$

55 $$K = \left[ {\begin{array}{*{20}c} {L_{1} } & 0 & 0 & 0 & 0 & 0 \\ 0 & {L_{2} } & 0 & 0 & 0 & 0 \\ 0 & 0 & {L_{3} } & 0 & 0 & 0 \\ 0 & 0 & 0 & {C_{1} } & 0 & 0 \\ 0 & 0 & 0 & 0 & {C_{2} } & 0 \\ 0 & 0 & 0 & 0 & 0 & {C_{o} } \\ \end{array} } \right]$$

56 $$A_{ = } \left[ {\begin{array}{*{20}c} 0 & 0 & 0 & {\left( {1 - D} \right)} & 0 & 0 \\ 0 & 0 & 0 & D & { - \left( {1 - D} \right)} & 0 \\ 0 & 0 & 0 & D & D & { - 1} \\ {\left( {1 - D} \right)} & { - D} & { - D} & 0 & 0 & 0 \\ 0 & {\left( {1 - D} \right)} & { - D} & 0 & 0 & 0 \\ 0 & 0 & { - 1} & 0 & 0 & { - \frac{1}{{R_{O} }}} \\ \end{array} } \right]$$

57 $$B = \left[ {\begin{array}{*{20}l} { - V_{C1} } \hfill & 1 \hfill \\ {V_{C1} + V_{C2} } \hfill & 0 \hfill \\ {V_{C1} + V_{C2} } \hfill & 0 \hfill \\ { - I_{L1} - I_{L2} - I_{L3} } \hfill & 0 \hfill \\ { - I_{L1} - I_{L3} } \hfill & 0 \hfill \\ 0 \hfill & 0 \hfill \\ \end{array} } \right]$$

The *V*_*o*_ matrices can be obtained:

58 $$V_{o} = \left[ {\begin{array}{*{20}c} 0 & 0 & 0 & 0 & 0 & 1 \\ \end{array} } \right]\left[ {\begin{array}{*{20}c} {\mathop {i_{L1} }\limits^{ \wedge } } \\ {\mathop {i_{L2} }\limits^{ \wedge } } \\ {\mathop {i_{L3} }\limits^{ \wedge } } \\ {\mathop {v_{C1} }\limits^{ \wedge } } \\ {\mathop {v_{C2} }\limits^{ \wedge } } \\ {\mathop {v_{Co} }\limits^{ \wedge } } \\ \end{array} } \right] + \left[ {\begin{array}{*{20}c} 0 & 0 \\ \end{array} } \right]\left[ {\begin{array}{*{20}c} {\mathop d\limits^{ \wedge } } \\ {\mathop {V_{in} }\limits^{ \wedge } } \\ \end{array} } \right]$$

**Figure 15:**
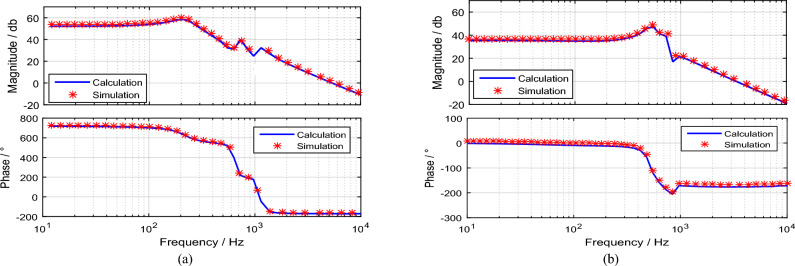
Bode diagrams (simulation and theoretical results comparison). (**a**) Boost operation; (**b**) Buck operation.

**Figure 16:**
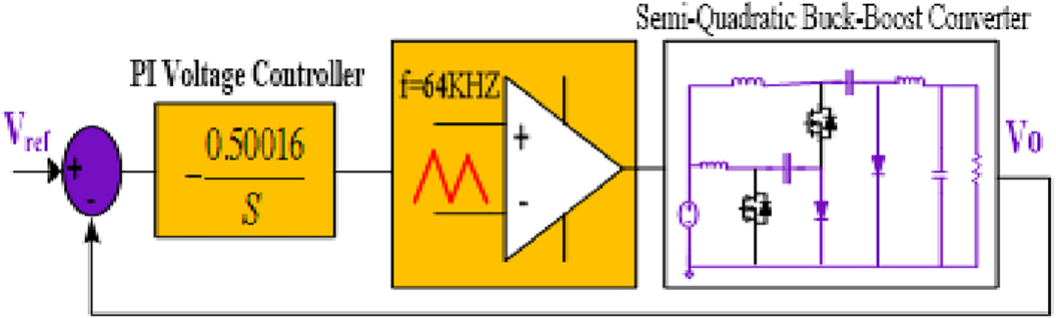
PI controller system for the proposed structure1.

To verify the model, the extracted bode diagrams from the calculation process and simulations are illustrated to control the output transfer function (Fig. 15). The parameters in Table 3 are utilized to sketch the bode diagrams. As seen, there is a good consistency between the calculated model and the results of the simulation. Therefore, the proposed model can be utilized for precise controller design and dynamic analysis. Based on the gain and phase margins, Fig. 16 shows the control system parameters using the SISOTOOL toolbox in MATLAB. The PI controller can control the *V*_*o*_ at 72 V and 15 V in step-up and step-down modes. Nevertheless, when the load is altered in an open-loop control system, the ripples in the output voltage change.

**Figure 17:**
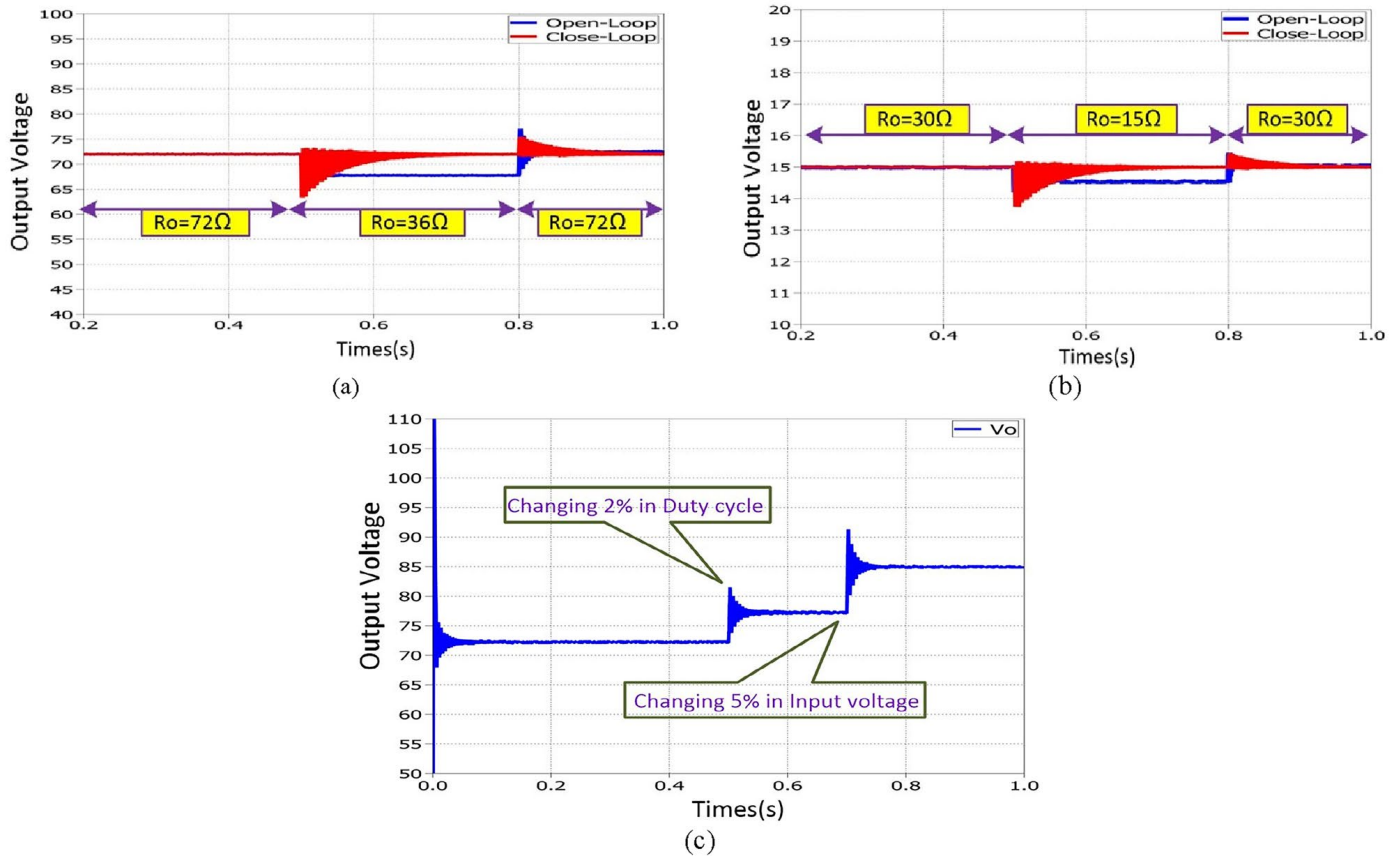
Dynamic response of proposed structure1, (**a**) Output voltage per change in output load resistance for constant input voltage (step-up mode) (**b**) Output voltage per change in output load resistance for constant input voltage (step-down mode); (**c**) Output voltage for small step change in duty cycle (2%) and input voltage (5%).

The small-signal dynamic response of the suggested converter for given perturbations in duty cycle, control input, input voltage and change in output load resistance is plotted in Fig. 17. The suggested converter has a good dynamic behavior over a small perturbation.

## Comparison with existing converters

Table 1 the comparison of structure 1 and 2 is presented. An overview of the suggested semi-quadratic converters and other existing converters is shown in Table 2. As observed, semiconductor element number was evaluated as well as switches’ voltage stress, voltage gain ratio, *SDP*_*avg*_*/P*_*o*_, output polarity, voltage stress of diodes, and continuous output/input current port for the presented converter and other converters. Although the presented structures in this case have a continuous input current and fewer semiconductor elements, it can certainly be a suitable option for PV inverters due to its continuous input current. With ten elements, high voltage gain ratios and the designed configuration structure1 includes continuous input/output current ports. The superiority of the presented structures is shown as below:

**Table 1:** Comparison of the proposed structures.

Item	Topology	
	Structures	
	Structure1	Structure2
Continuous Input/output	✓/✓	✓/ ×
Output Polarity	Negative	Positive
V_C2_ /V_in_	$1/(1 - D)^{2}$	1

**Table 2:** Evaluation of the suggested structures and the existing converters.

Topology	Item											
	Number of Elements					Voltage Gain	V_S_/V_in_	V_D_/V_in_	Norm SDP_avg_ /P_o_	V_C_/V_in_	Output Polarity	Continuous Input/output
	S	D	C	L	T							
Proposed Structure1	2	2	3	3	**10**	$\frac{D(2-D)}{{(1-D)}^{2}}$	$\frac{1}{1-D}\frac{1}{(1-D{)}^{2}}$	$\frac{1}{1-D}$ $\frac{2-D}{(1-D{)}^{2}}$	$\frac{2}{D(1-D)(2-D)}$	$\frac{1}{1-D}$ $\frac{1}{(1-D{)}^{2}}$	Negative	Yes/Yes
Proposed Structure2	2	2	3	3	**10**	$\frac{D(2-D)}{{(1-D)}^{2}}$	$\frac{1}{1-D}\frac{1}{(1-D{)}^{2}}$	$\frac{1}{1-D}$ $\frac{2-D}{(1-D{)}^{2}}$	$\frac{2}{D(1-D)(2-D)}$	$\frac{1}{(1-D{)}^{2}}$ 1	Positive	Yes/No
IN^41^	2	2	3	3	**10**	$\frac{{D}^{2}}{(1-D{)}^{2}}$	$\frac{1}{1-D}\frac{D}{(1-D{)}^{2}}$	$\frac{1}{1-D}\frac{D}{(1-D{)}^{2}}$	$\frac{{D}^{2}-D+2}{D(1-D)}$	$\frac{1}{1-D}$ $\frac{2D - 1}{{(1 - D)^{2} }}$	Positive	Yes/Yes
IN^39^	1	5	3	3	**12**	$\frac{{D}^{2}}{(1-D{)}^{2}}$	$\frac{1}{(1-D{)}^{2}}$	$\frac{1}{1-D}$ $\frac{D}{(1-D{)}^{2}}$ $\frac{1}{1-D}$ $\frac{1}{1-D}$ $\frac{D}{(1-D{)}^{2}}$	$\frac{6{D}^{2}-8D+4}{D(1-D{)}^{2}}$	$\frac{1}{1-D}$ $\frac{1}{(1-D{)}^{2}}$	Negative	Yes/Yes
IN^38^	2	2	3	3	**10**	$\frac{{D}^{2}}{(1-D{)}^{2}}$	$\frac{1}{(1-D{)}^{2}}$ $\frac{D}{(1-D{)}^{2}}$	$\frac{1}{1-D}$ $\frac{D}{(1-D{)}^{2}}$	$\frac{2}{D(1-D)}$	$\frac{1}{1-D}$ $\frac{D}{(1-D{)}^{2}}$	Positive	Yes/Yes
IN^37^	2	2	3	3	**10**	$\frac{{D}^{2}}{(1-D{)}^{2}}$	$\frac{1}{1-D}$ $\frac{D}{(1-D{)}^{2}}$	$\frac{1}{1-D}$ $\frac{D}{(1-D{)}^{2}}$	$\frac{1+D}{D(1-D)}$	$\frac{1}{1-D}$ $\frac{D}{(1-D{)}^{2}}$	Positive	Yes/Yes
IN^36^	2	2	3	3	**10**	$\frac{D}{(1-D{)}^{2}}$	$\frac{1}{1-D}$ $\frac{1}{(1-D{)}^{2}}$	$\frac{1}{(1-D{)}^{2}}$ $\frac{D}{(1-D{)}^{2}}$	$\frac{1+D}{D(1-D)}$	$\frac{1}{(1-D{)}^{2}}$ $\frac{D}{(1-D{)}^{2}}$	Negative	Yes/Yes
IN^35^	2	4	2	4	**10**	$\frac{D}{(1-D{)}^{2}}$	$\frac{1}{(1-D{)}^{2}}$	1 $\frac{D}{(1-D{)}^{2}}\frac{{D}^{2}}{(1-D{)}^{2}}$				
$\frac{D}{1-D}$	$\frac{2{D}^{3}-{D}^{2}+1}{D(1-D{)}^{2}}$	$\frac{D}{1-D}$	Positive	Yes/No								
IN^34^	2	2	2	2	**8**	$\frac{D}{(1-D{)}^{2}}$	$\frac{1}{1-D}\frac{1}{(1-D{)}^{2}}$	$\frac{1}{1-D}$ $\frac{1}{(1-D{)}^{2}}$	$\frac{1+D}{D(1-D)}$	$\frac{1}{1-D}$	Positive	Yes/No
IN^33^	1	2	4	3	**10**	$\frac{2D}{1-D}$	$\frac{1}{1-D}$	$\frac{1}{1-D}\frac{1}{1-D}$	$\frac{2}{D(1-D)}$	$\frac{2D}{1-D}\frac{D}{1-D}$ $\frac{D}{1-D}$	Positive	No/Yes
IN^32^	1	2	4	3	**10**	$\frac{2D}{1-D}$	$\frac{1}{1-D}$	$\frac{1}{1-D}\frac{1}{1-D}$	–––––––-	1 $\frac{D}{1-D}$ $\frac{D}{1-D}$	Positive	Yes/Yes
IN^31^	1	3	5	3	**12**	$\frac{3D}{1-D}$	$\frac{1}{1-D}$	$\frac{1}{1-D}$ $\frac{2D}{1-D}$ $\frac{1}{1-D}$	$\frac{2}{D(1-D)}$	$\frac{2D}{{1 - D}}$		
$\frac{D}{1-D}\frac{D}{1-D}$ $\frac{2D}{1-D}$	Negative	No/No										
IN^30^	1	3	6	4	**14**	$\frac{3D}{1-D}$	$\frac{1}{1-D}$	$\frac{1}{1-D}\frac{1}{1-D}$ $\frac{1}{1-D}$	–––––––-	––––––––	Positive	Yes/No
IN^29^	2	2	2	2	**8**	$\frac{D(2-D)}{{(1-D)}^{2}}$	$\frac{1}{1-D}$ $\frac{1}{(1-D{)}^{2}}$	$\frac{1}{1-D}$ $\frac{1}{(1-D{)}^{2}}$	$\frac{2}{D(1-D)(2-D)}$	$\frac{1}{1-D}$	Negative	No/No
CUK Converter	1	1	2	2	**6**	$\frac{D}{1-D}$	$\frac{1}{1-D}$	$\frac{1}{1-D}$	–––––––-	$\frac{1}{1-D}$	Negative	Yes/Yes

### Voltage gains comparison

**Figure 18:**
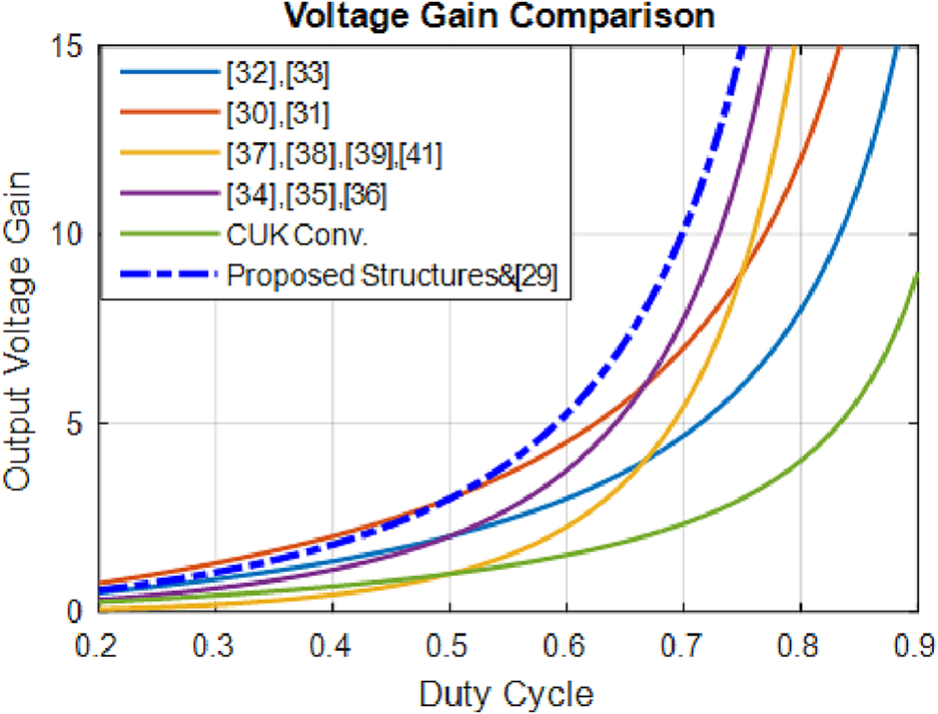
Plot of output voltage gain versus duty cycles.

Comparing the voltage gain ratios is shown in Fig. 18, while Table 2 lists competitor converters and the data they provide. The considered converter provides a larger voltage gain than similar converters when *D* > *0.5*. As seen, the horizontal axis indicates the duty cycle (20–90%). Furthermore, the vertical axis represents the output voltage over the range of 0-15 V. The higher *D* and *V*_*o*_, but at the expense of more loss of power.

### Effectiveness index

Here, the proposed structures performance is assessed with other buck/boost converters for validating the aforementioned properties. In Table 2, a detailed comparative analysis is presented based on the maximum voltage stress on diodes, common ground characteristics, voltage gain, input/output current ripple, the voltage stress on switches, and the overall number of elements. Furthermore, an efficiency index (EI) is presented for evaluating the ratio between the whole number of used elements and the voltage gain value^22,23^. EI is calculated as follows:

59 $$EI = \frac{{\frac{{D\left( {2 - D} \right)}}{{\left( {1 - D} \right)^{2} }}}}{Total\,Number\, of\, Utilized\, Elements }$$

**Figure 19:**
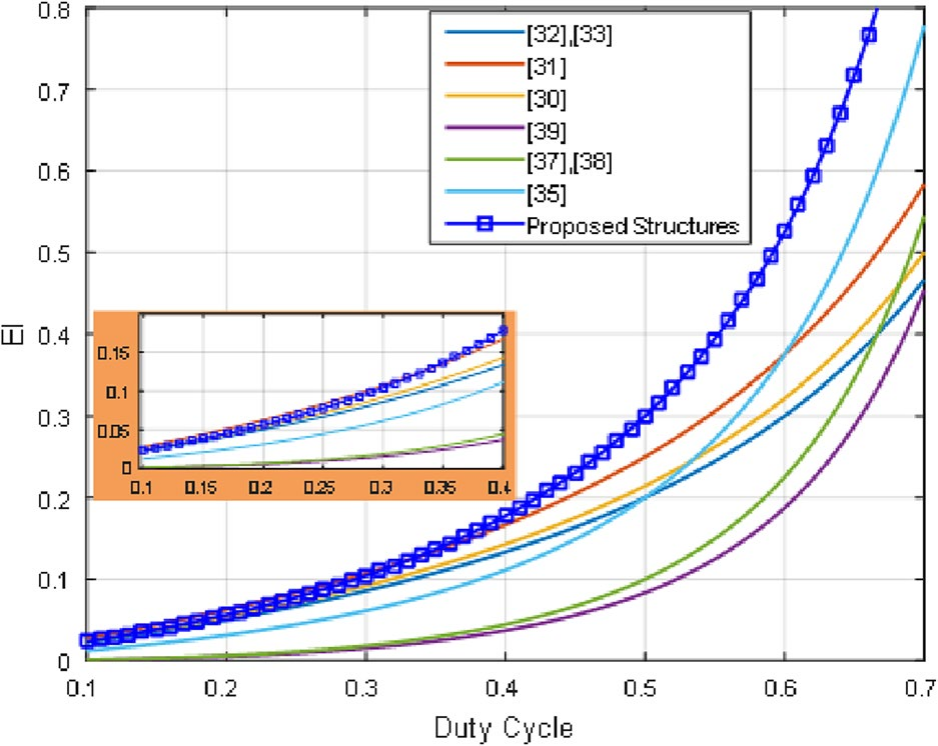
Effectiveness index.

This parameter accurately depicts the converter’s power density and optimal circuit element use. Figure 19 shows the EI plotted against duty cycle for the converter that is being shown and several comparable converters.

### Voltage stress on elements

**Figure 20:**
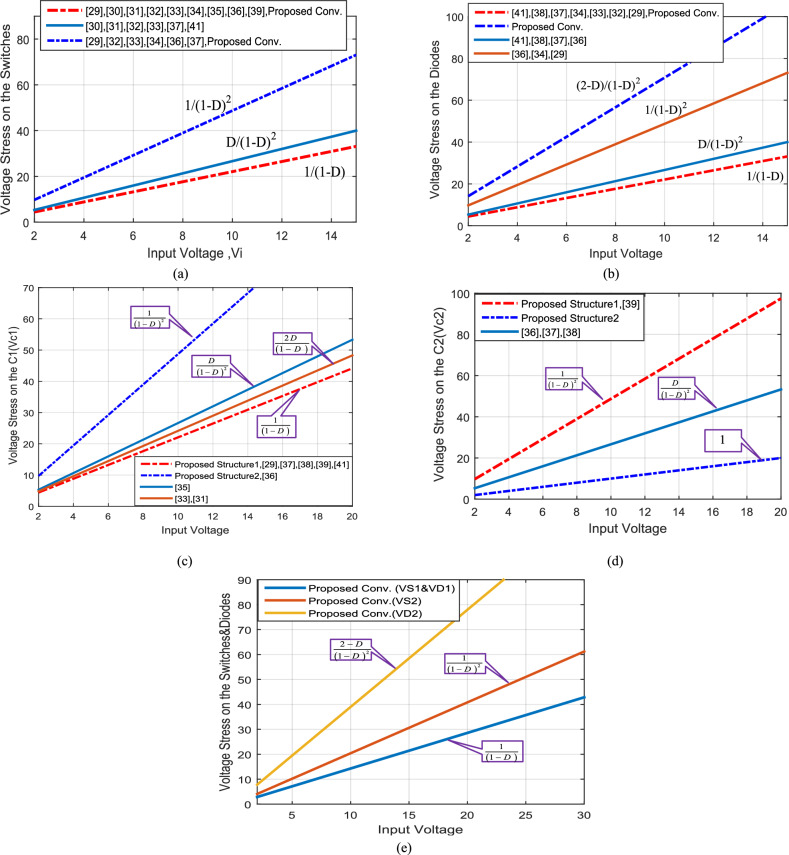
Comparison of Voltage stress, (**a**) Voltage stress on the switches; (**b**) Voltage stress on the diodes; (**c**) Voltage stress on the capacitor C_1_; (**d**) Voltage stress on the capacitor C_2_; (**e**) Voltage stress on the switches and diodes of the proposed structures.

As shown in Fig. 20a the electrical voltage stress on the semiconductor switches *S*_*1*_ and *S*_*2*_ for different input voltage. In Fig. 20b, the voltage stress on diodes *D*_*1*_ and *D*_*2*_ are compared. I can be seen, that the voltage stress on the switch *S*_*2*_ and diode *D*_*2*_ of the proposed converter is higher than other similar converters, but finally the switching device power (SDP) of proposed converter is lower than other converters as shown in next section. Additionally, in most conventional topologies, the voltage stress on capacitors *C*_*1*_ and *C*_*2*_ is identical, and hence, the result can be observed from Fig. 20c and d, where nearly all topologies have the close and same voltage stress on capacitors *C*_*1*_ and *C*_*2*_. In Fig. 20e, the voltage stress on switches and diodes of the proposed structures is shown.

### Total SDP

*SDP* is an appropriate index to evaluate different features of switching devices like cooling system necessities, potential costs, and losses^48,52,53^. When evaluating the power loss and the ultimate cost of implementation of a converter, voltage stress and current of its switches and diodes are considered. Therefore, it is necessary to investigate such stresses. As discussed previously, *SDP* can help calculate the cost and power loss of the converter. The overall average of *SDP* (*SDP*_*avg*_) is determined as follows:

60 $$SDP_{avg} = V_{S1} I_{S1 - avg} + V_{S2} I_{S2 - avg} + V_{D1} I_{D1 - avg} + V_{D2} I_{D2 - avg}$$

**Figure 21:**
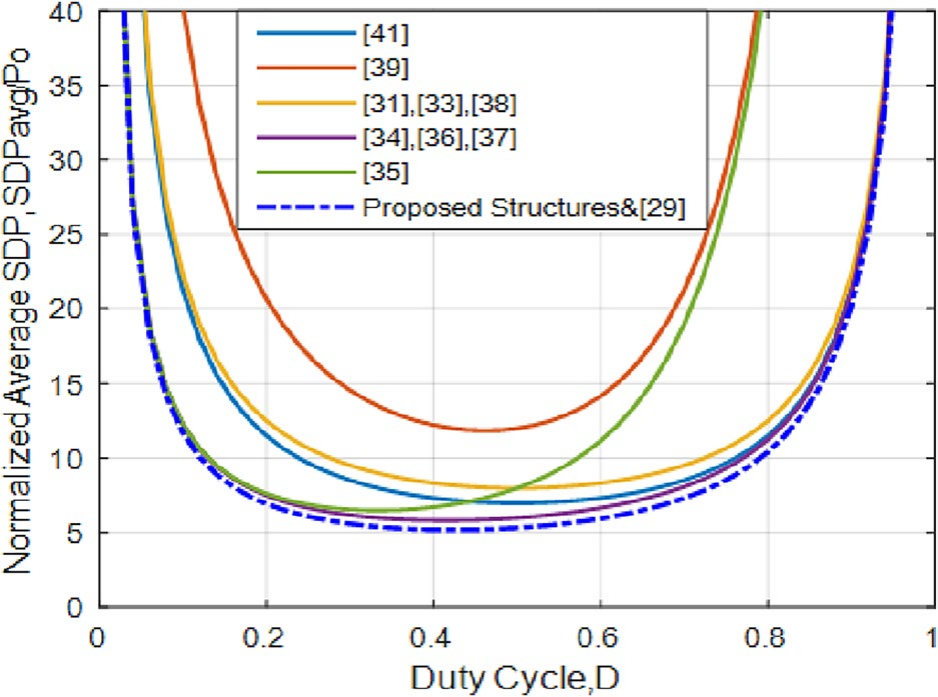
Plot of normalized SDP_avg_ vs duty cycle.

In which, *I*_*Savg_i*_ and *V*_*S_i*_ denote the average current and peak voltage of a switching period of the i^th^ semiconductor of the converter. For different converters, the total average *SDP*s is represented in Fig. 21. Compared to similar buck/boost converters, the presented converter achieves lower *SDP*, which directly leads to lower semiconductor costs and power losses. During buck/boost operation, the total average *SDP* is determined as follows:

61 $$SDP_{avg} = 2P_{o} /D\left( {1 - D} \right)\left( {2 - D} \right)$$

## Experimental result and building a prototype

Experimental simulations and examinations of the presented buck-boost structure1 proved the validity of the theoretical models and experiments for both operation modes.

**Figure 22:**
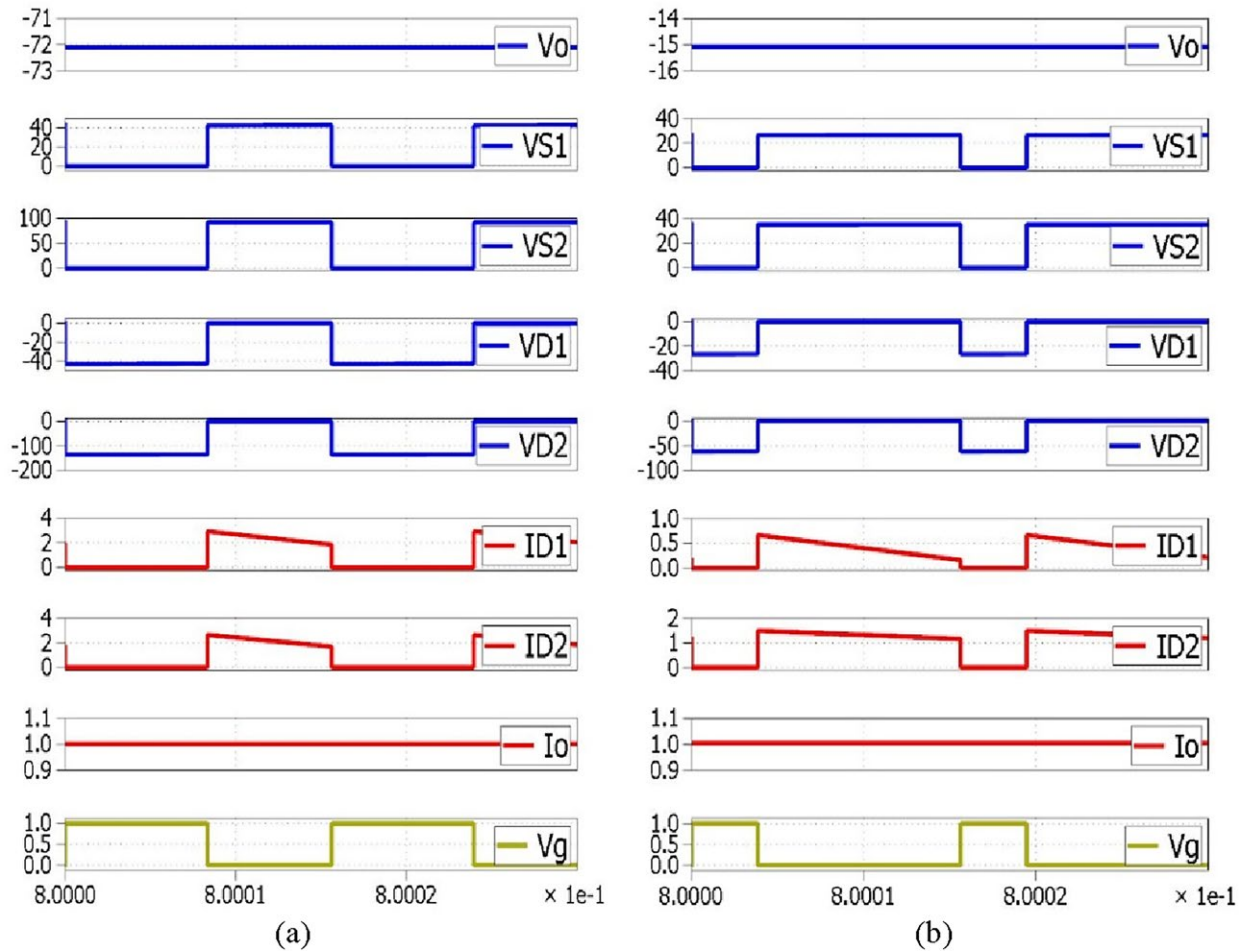
Simulated waveforms of in PLECS. (**a**) Boost operation; (**b**) Buck operation.

A list of the elements used in simulation and experiments on the presented converter is shown in Table 3. These parameters were selected using Eqs. (44)–(46) and (33)–(35). The current variations of inductors were set at 30%, and the voltage ripples of capacitors *C*_*o*_*, C*_*2*_ and *C*_*1*_ were regulated at 0.5%, 2% and 2%, respectively. The ripples of capacitor voltage were considered to calculate the capacitance of *C*_*o*_*, C*_*2*_ and *C*_*1*_ using Eqs. (44)–(46). The inductance of *L*_*3*,_ *L*_*2*_ and *L*_*1*_ was selected in terms of the inductor current ripples (33)–(35). When the converter is operating in step-up mode, its voltage input level can be adjusted within the range of 20 V dc to 72 V dc and with negative polarity. The inductor current waveforms for *L*_*3*,_ *L*_*2*_ and *L*_*1*_ are displayed in the CCM mode along with the converter input continuous current. Furthermore, considering the input voltage of 20 V and duty cycle of 0.534, the mean current of inductors *L*_*3*,_ *L*_*2*_ and *L*_*1*_ is 1 A, 1.13 A, and 2.52 A respectively. Based on Eqs. (5) and (6), the voltage of capacitors *C*_*o*_, *C*_*2*_ and *C*_*1*_ is around 71.09 V, 92.09 V, and 42.80 V, respectively. The power switches *S2, S*_*1,*_ and diodes *D*_*2*_ and* D*_*1*_ were selected according to the current stress and voltage of the components. The mean voltages stress for these four components was 92.22 V, 42.91,  − 135.26 V, and  − 43.4when calculating the type of switch and diode. The average current of inductors *L*_*3*,_ *L*_*2*_ and *L*_*1*_ is 1A, 0.32A, and 0.41A, respectively in step-down mode, by the input voltage of 20 V and the duty cycle of 0.245. Furthermore, the quantity of voltage stress applied on capacitors* C*_*o*_, *C*_*2*_ and *C*_*1*_ is 14.98 V, 35.07 V, and 26.47 V, respectively. Besides, based on the analytic equations, the voltage stresses applied on switches and diodes are 26.41 V, 20.01 V,  − 61.71 V, and  − 26.58 V, respectively. Figure 22 shows the simulation waveforms of the structure1 under CCM mode operation via the PLECS in the boost and buck operation. It represents the current and output voltage, gate- source voltage, diodes voltage and current, and switch voltage of the MOSFET.

**Table 3:** Experimental setup parameters. Significantvalues in bold.

Parameters	Prototype	
	Boost operation	Buck operation
*Duty Cycle*	0.534	0.245
*Vo*	**72 V**	**15 V**
*Vin*	20 V	20 V
*fs*	64KHZ	
*D*_*1*_*/D*_*2*_	SR5200 (*V*_*FD*_ ~ 0.9 V)	
*S*_*1*_*/S*_*2*_	IRFP4668PBF (*R*_*DS*_ = 8mΩ) *t*_*on*_ = 41 ns*, t*_*off*_ = 4 ns,*Coss* = 810PF	
*C*_*1*_*/C*_*2*_*/C*_*o*_ *R*_*C1*_*/R*_*C2*_*/R*_*Co*_	33µF/ 33µF/ 64 µF 0.021Ω/0.022Ω/0.027Ω	
*L*_*1*_*/L*_*2*_*/L*_*3*_ *R*_*L1*_*/R*_*L2*_*/R*_*L3*_	152µH/810µH/1.7mH 0.11Ω/0.21Ω/0.10Ω	
***Drive Circuit*** *R1 / R2/ Cn*_*1*_* /Cn*_*2*_	1.2KΩ/560Ω/104nF /104nF	
Height /Width / Length (Prototype Made): 3.4 cm /11.5 cm /7.5 cm		
Weight Prototype Made: 210 g

## Building a prototype and performance evaluation

**Figure 23:**
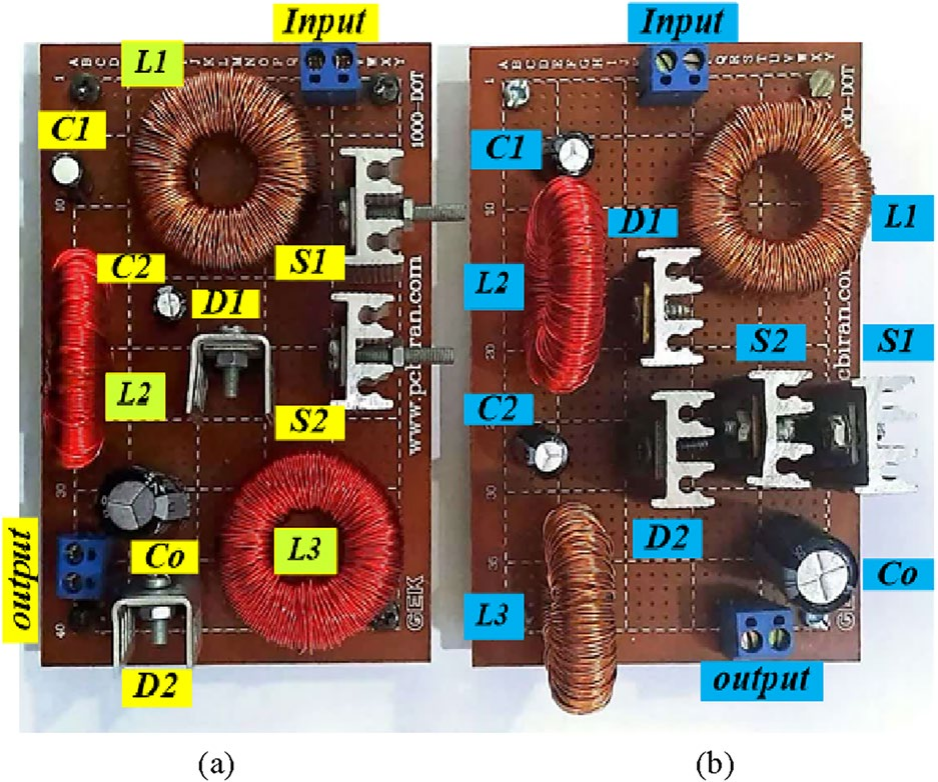
Hardware prototype. (**a**) Structure 2; (**b**) Structure 1.

**Figure 24:**
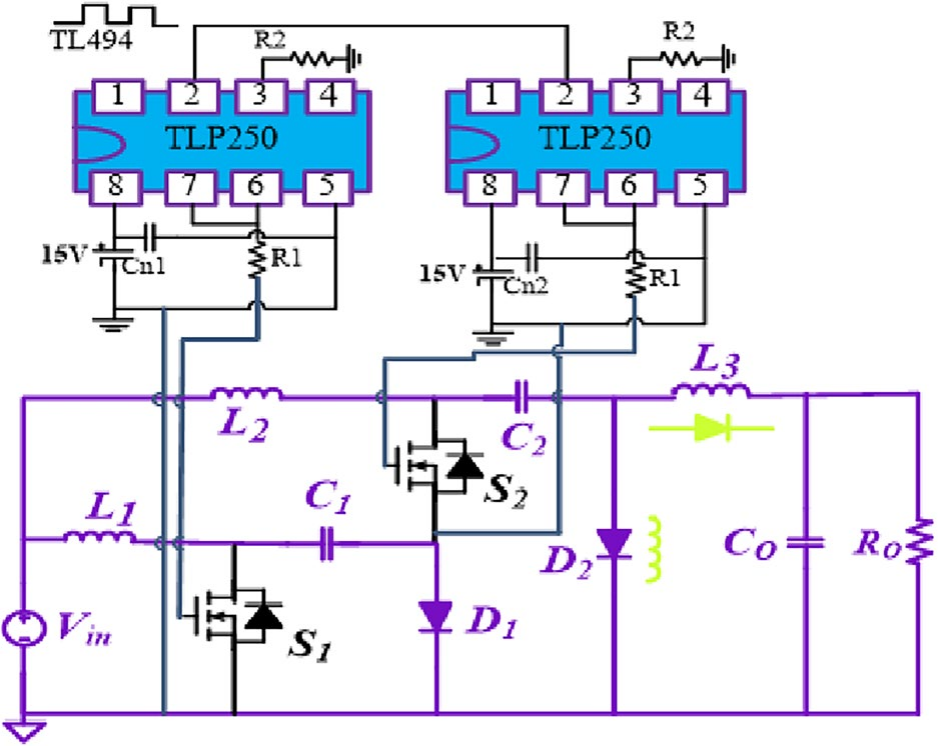
Driving MOSFET Circuit.

Here, to verify the aforesaid properties of the suggested structures, an experimental prototype is used. The prototype was run and examined with 15W (buck mode) and 72W (boost mode) output powers. Figures 23 and 24 show the experimental prototype and driving circuit. The circuit elements corresponding to the experimental converter are presented in Table 3. According to Table 3, the power switches are two MOSFETs (IRFP4668PBF). SR5200 diodes were selected for realizing the diodes. The photocoupler TLP250 was utilized to drive the switches S_2_ and S_1_.

**Figure 25:**
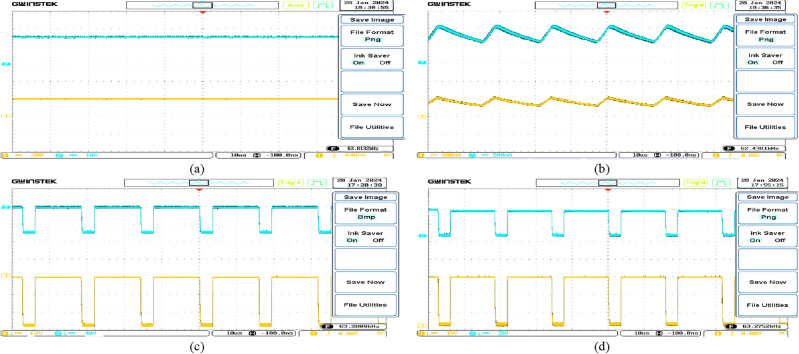
Experimental waveforms in the buck mode (structure1); (**a**) Vin (20 V/div), Vo(10 V/div); (**b**) i_L1_(500 mA/div), i_L3_(500 mA/div); (**c**) V_D1_(10 V/div), V_D2_(40 V/div); (**d**) V_S1_(10 V/div), V_S2_(20 V/div).

**Figure 26:**
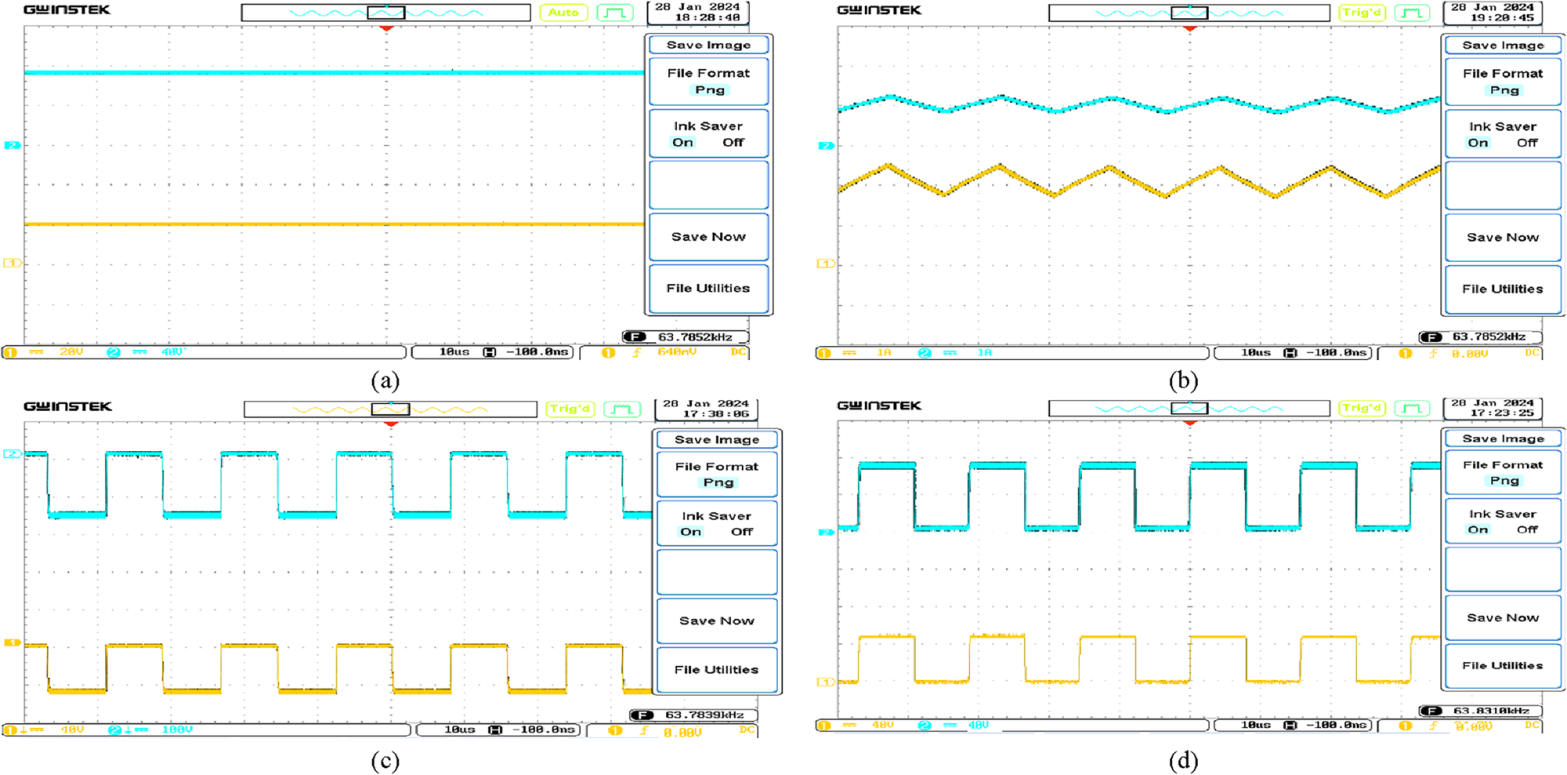
Experimental waveforms in the boost mode (structure1); (**a**) Vin (20 V/div), Vo(40 V/div); (**b**) i_L1_(1A/div), i_L3_(1A/div); (**c**) V_D1_(40 V/div), V_D2_(100 V/div); (**d**) V_S1_(40 V/div), V_S2_(40 V/div).

**Figure 27:**
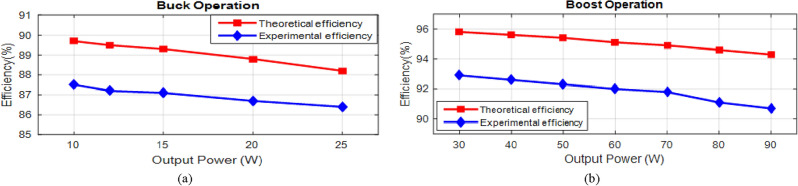
The efficiency of Experimental and theoretical result. (**a**) Buck Operation; (**b**) Boost Operation.

**Figure 28:**
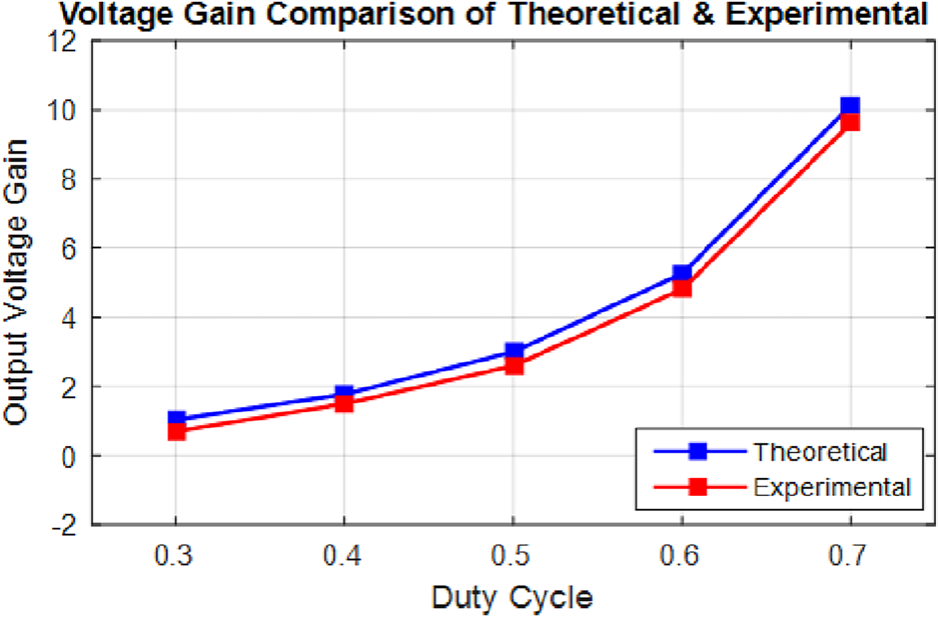
Voltage gain ratio comparison of theoretical and experimental results.

The experimental waveforms of the experimental prototype are shown in Figs. 25 and 26 (structure 1), which were recorded and measured via a GW INSTEK GDS-2102A oscilloscope, GW INSTEK GPS-4303 power supply and a Pintek PA- 667 1 MHz current probe. Figures 25 and 26 represent the converter operation and the accuracy of the mathematical model via the experimental prototype. Moreover, the waveforms of the voltages and currents of the intended converter in dual modes are displayed. These standards were measured in the laboratory. Results from the PLECS simulations and experiments differed very slightly. Finally, the test results proved that the operation of the converter as presented is completely valid, verifying the principles of process and features outlined earlier. The experimental and theoretical proficiency curves of the proposed converter are displayed for varying output power in Fig. 27a and b for buck and boost operation respectively. A theoretical and experimental analysis of buck and boost operation modes is shown in Table 4 (structure1). A voltage gain for the presented converter is presented in Fig. 28 based on theoretic and experimental outcomes. Voltage gain values achieved experimentally differ from theoretical values due to the parasitic parameters of different circuit elements.

**Table 4:** Theoretical and experimental results comparison.

Symbol	Boost Operation		Buck Operation	
	Theo	Exp	Theo	Exp
***D***	0.534	0.542	0.245	0.258
***ΔV***_***C1***_	0.56	0.62	0.16	0.18
***ΔV***_***C2***_	0.26	0.28	0.12	0.12
***ΔI***_***L1***_	1.09	1.23	0.53	0.60
***ΔI***_***L2***_	0.64	0.72	0.23	0.29
***ΔI***_***L3***_	0.30	0.39	0.11	0.15

Theo = Theoretical, Exp = Experimental.

Significant values in bold.

## Conclusion

In this paper, two new transformerless DC/DC buck/boost converters for PV application are introduced. So the features proposed structures has semi-quadratic type buck/boost conversion, common ground, minimal ripples comparable to those in the Cuk converter at output and output sides (structure1), high voltage gain, and negative and positive output load voltage polarity for structures1and 2 respectively. The operating principle of the presented structures is described and steady-state analysis is performed to obtain the voltage and current relations in the two modes of operation. The efficiency analysis and power loss estimation are carried out considering parasitic elements of the converter. The design of passive elements based on the ripple voltages and currents is discussed. The developed structures performance is evaluated in comparison with similar existing converters and is observed to possess a higher voltage gain ratio, high efficiency, and small SDP than other similar converters. To test and verify the performance of the converter, it has been adjusted at various duty cycle values. The peak efficiency of the converter was 91.8% at 72 W. Considering the features of the designed converter including the continuous output/input current port and negative output polarity characteristics, it can be considered appropriate for applications including renewable energy, multi-function power supplies, signal generators and audio amplifiers.

## Data Availability

Upon reasonable request, the corresponding author will make the datasets used in this study available.
